# Hemi‐Indigiosin: A pH and Red‐Light Responsive Transmembrane HCl Transporter

**DOI:** 10.1002/anie.202515930

**Published:** 2025-10-30

**Authors:** Nol Duindam, Jasper E. Bos, Felix van Nifterik, Sander J. Wezenberg

**Affiliations:** ^1^ Leiden Institute of Chemistry Leiden University Einsteinweg 55 Leiden 2333 CC The Netherlands

**Keywords:** Anion transport, Hemi‐indigo, Lipid bilayers, Molecular switches, Photochromism

## Abstract

Synthetic anion transporters have emerged as promising therapeutic agents. However, their targeting ability is still low. Herein, we present hemi‐indigiosin, which is structurally related to prodigiosin—a well‐studied natural product exhibiting a wide range of biological activities. Our hemi‐indigiosin is shown to have similar HCl transport selectivity and pH‐dependent activity as prodigiosin, while in addition it can be deactivated by irradiation with visible light. This deactivation is effective due to high photoconversion from the active chloride‐binding *Z*‐isomer to the inactive *E*‐isomer, not exposing a chloride‐binding site. Transport activity is thus controlled by both pH change and light, which will allow improved targeting of pathological sites and prevent ecological impact after drug excretion into the environment.

## Graphical abstract

A hemi‐indigo‐based transmembrane HCl transporter is presented, showing similar selectivity and pH‐responsivity as the natural product prodigiosin. In addition, visible‐light irradiation of the active *Z*‐isomer gives high conversion to the inactive *E*‐isomer. This light response could diminish undesired side effects in potential therapeutic applications and avoid ecological impact after excretion of the molecule into the environment.

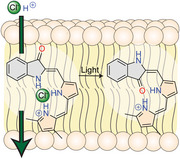

Artificial molecular systems that facilitate passive anion transport across biological membranes have emerged as promising therapeutic agents.^[^ ^1^, ^2^, ^3^ ^]^ These systems may one day be used to take over the function of malfunctioning transport proteins as therapy for channelopathies^[^ ^4^, ^5^, ^6^ ^]^ while, furthermore, their ability to trigger cell death through disruption of ion homeostasis could be leveraged for anticancer or antibacterial treatment.^[^ ^7^, ^8^, ^9^, ^10^ ^]^ In the pursuit of novel anticancer agents, the natural product prodigiosin, a remarkably active HCl transporter,^[^ ^11^, ^12^, ^13^ ^]^ inspired the design of many new transporters, including tambjamines,^[^ ^8^ ^]^ obatoclax,^[^ ^14^ ^]^ and perenosins.^[^ ^15^, ^16^ ^]^ For the latter compound class, like for prodigiosin,^[^ ^11^ ^]^ higher transport activity was observed at slightly acidic pH, which could impose selectivity toward cancer cells (tumors have an acidic microenvironment).^[^ ^1^, ^2^, ^3^, ^15^, ^16^ ^]^ Also, other transporters responsive to pH, either based on binding site (de)protonation,^[^ ^17^, ^18^, ^19^, ^20^, ^21^, ^22^ ^]^ or conformational changes^[^ ^23^, ^24^ ^]^ have been reported. Nevertheless, poorly targeted cytotoxicity of anticancer drugs causes undesirable side effects and, after excretion of the drug, negative environmental effects persist. That is, anticancer drugs in wastewater pose a potential ecological risk.^[^ ^25^ ^]^

The incorporation of molecular photoswitches into drugs has proven promising to control biological activity on demand with high spatiotemporal precision.^[^ ^26^, ^27^ ^]^ This approach can be used to reduce undesired drug activity away from the pathological site, as well as to diminish environmental toxicity. For example, photoswitchable antibiotics have been developed to combat antimicrobial resistance,^[^ ^28^, ^29^ ^]^ and photoswitchable anticancer agents have shown potential as precision‐targeted chemotherapeutics.^[^ ^30^ ^]^ In recent years, a similar strategy has been applied to synthetic anionophores to switch between states with high and low transport activity.^[^ ^31^, ^32^, ^33^, ^34^, ^35^, ^36^, ^37^, ^38^ ^]^ However, in order to achieve complete deactivation upon light irradiation, quantitative conversion to an inactive isomer is required, which poses a major challenge. Most light‐responsive transporters developed so far maintain a significant amount of active form at photoequilibrium. Also, as they commonly rely on a photoswitchable core interconnecting two binding units (viz., molecular tweezers), a binding site is exposed in the low activity form, leading to considerable residual transport. Furthermore, UV light is most frequently used to induce the photoresponse, limiting biological application as it is damaging to cells and has low tissue penetration depth.^[^ ^26^, ^27^ ^]^

Hence, we envisioned a transporter in which the binding site is blocked by intramolecular interactions in one state,^[^ ^39^, ^40^, ^41^ ^]^ where photoconversion to this state is nearly quantitative.^[^ ^42^ ^]^ These requirements led us to explore the hemi‐indigo scaffold, as it already contains an NH hydrogen bond donor, and its *Z*‐isomer can be adapted to resemble prodigiosin when functionalized with an appropriate dipyrrin moiety (Scheme 1a). Photoisomerization to the *E*‐isomer, induced by visible light, would block the dipyrrin binding motif through intramolecular hydrogen bonding with the carbonyl oxygen. Moreover, based on recent work on *N*‐heterocyclic hemi(thio)indigo dyes,^[^ ^43^, ^44^, ^45^, ^46^ ^]^ irradiation was expected to lead to high conversion to the intramolecularly hydrogen‐bonded species, irrespective of the visible‐light wavelength used. As an additional feature of interest, the envisioned light‐responsive prodigiosin analog was expected to show pH‐dependent activity, since protonation of the dipyrrin fragment is key in prodigiosin‐mediated HCl transport across the membrane.^[^ ^11^, ^12^, ^13^ ^]^ This type of transporter would thus be responsive to endogenous (pH) as well as exogenous (light) stimuli, and such dual responsivity could further increase the level of control over transport activity.

**Scheme 1:**
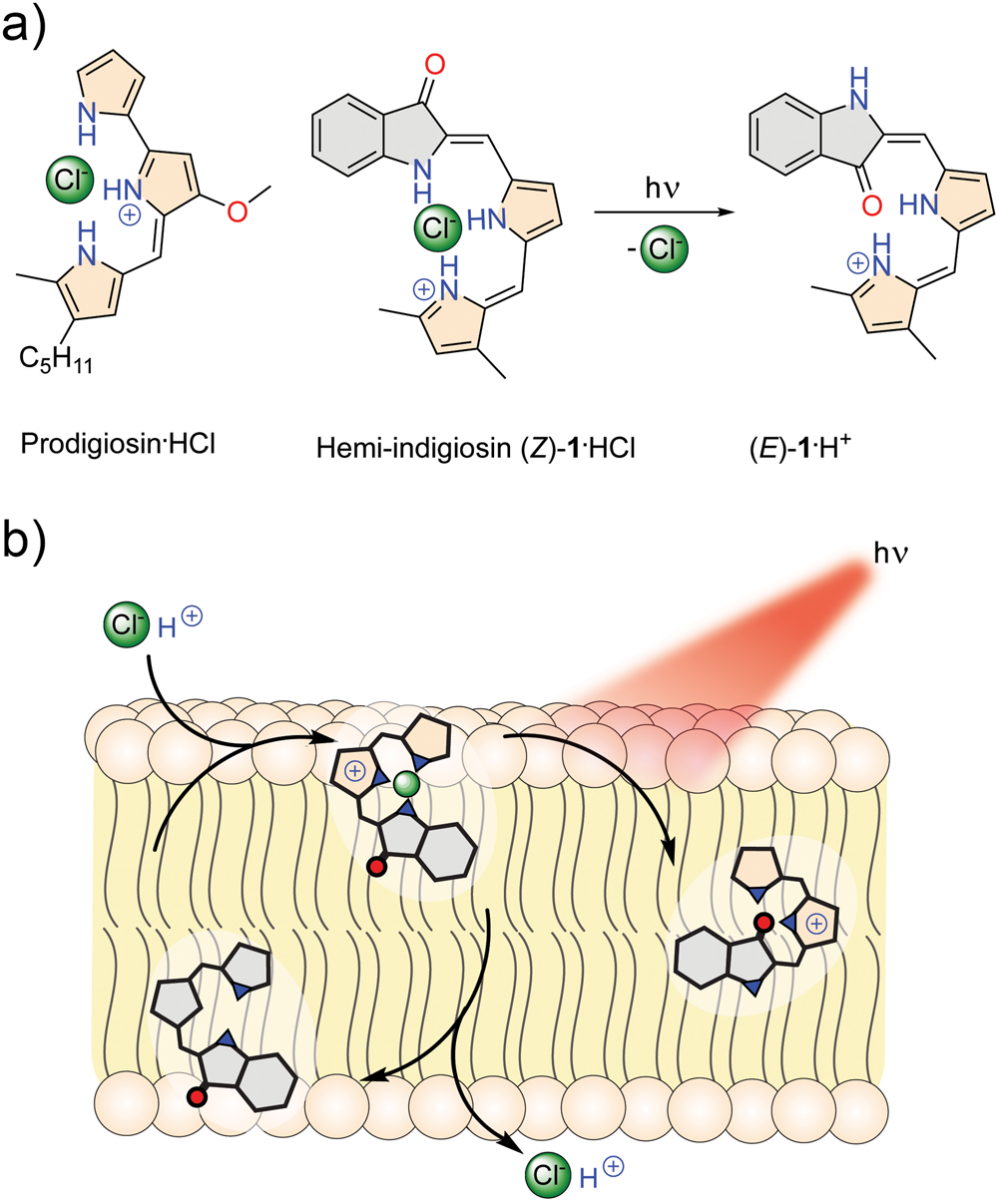
a) Structures of the HCl complexes of prodigiosin and the photoresponsive hemi‐indigiosin analog (*Z*)‐**1**, which undergoes *Z→E* isomerization in response to visible light. b) Schematic representation of electroneutral HCl transport across the bilayer membrane by (*Z*)‐**1** and deactivation of this transport by photoisomerization to (protonated) (*E*)‐**1**.

Herein we present hemi‐indigiosin (*Z*)‐**1**, which shows pH and red‐light responsivity (see Scheme 1b). It is found to be highly selective for electroneutral HCl over electrogenic Cl^−^ transport, with higher activity at lower pH values. Near quantitative *Z*→*E* isomerization is achieved upon irradiation with visible light, leading to deactivation of HCl transport. To our knowledge, this system represents the first visible‐light‐switchable transporter with demonstrated HCl selectivity. We envision the combined pH and red‐light responsivity to be an important step toward the development of anionophores into therapeutics with high targeting efficiency.

To validate the formation of a suitable chloride‐binding pocket in the *Z*‐isomer and intramolecular hydrogen bonding in the *E*‐isomer, DFT calculations were performed at the B3LYP‐D3/aug‐cc‐pVTZ level of theory using an IEFPCM MeOH solvation model. The energy‐minimized structure of (*Z*)‐**1** ^.^HCl shows three hydrogen bonds between the N─H protons and the chloride anion [N(H)^…^Cl distances of 3.142, 3.359, and 3.119 Å, Figures 1a and S43]. The lowest‐energy conformer of (*E*)‐**1** ^.^H^+^ displays two intramolecular hydrogen bonds between the N─H atoms of the protonated dipyrrin unit and the carbonyl oxygen of the indigo fragment [N(H)^…^O distances of 2.637 and 3.128 Å, Figures 1b and S44]. Also, the structure of (*Z*)‐**1** ^.^H^+^ was optimized, and its lowest‐energy conformer was found to be 20.6 kJ mol^−1^ higher in Gibbs free energy than that of (*E*)‐**1** ^.^H^+^ (Figure S45). These calculations thus confirm that a suitable binding site with three N─H hydrogen bond donors is available in the *Z*‐isomer, while interaction of chloride with the two N─H donors of the protonated dipyrrin moiety is restricted in the *E*‐isomer.

**Figure 1:**
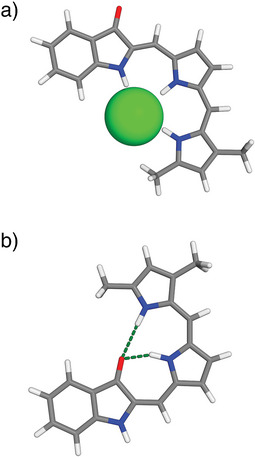
DFT energy‐minimized geometries (B3LYP‐D3/aug‐cc‐pVTZ, IEFPCM MeOH) of a) (*Z*)‐**1** ^.^HCl and b) (*E*)‐**1** ^.^H^+^ shown in stick representation (with chloride in space‐filling representation); color codes: hydrogen bond, Cl = green; O = red; N = blue.

Isomer (*Z*)‐**1** was then synthesized starting from the known 2‐pyrrole‐derived hemi‐indigo (Scheme S1).^[^ ^47^ ^]^ Synthetic routes toward dipyrrins often employ a MacDonald‐type condensation of a pyrrole fragment with 5‐formyl pyrrole in the presence of strong acid.^[^ ^48^ ^]^ In our case, however, such a procedure afforded the product in only trace amounts (Table S1), which is ascribed to the low nucleophilicity of the 2‐pyrrole‐derived hemi‐indigo precursor. Nevertheless, when in a different procedure, POCl_3_ was used to mediate the condensation,^[^ ^48^, ^49^, ^50^, ^51^ ^]^ the conversion improved. The product was isolated by precipitation followed by lyophilization, both in the presence of TFA, to obtain (*Z*)‐**1^.^** TFA in 62% yield as the TFA salt (as confirmed by ^19^F NMR spectroscopy and elemental analysis; see the Supporting Information).

The photoisomerization behavior was first studied by UV–vis spectroscopy. Therefore, the as‐isolated (*Z*)‐**1^.^** TFA salt was dissolved in MeOH, to which some TFA (12 equiv.) was added to ensure quantitative protonation (Figure S10).^[^ ^52^ ^]^ As can be observed in the spectrum in Figure 2, the compound absorbs in the visible light region (*λ*_max_ = 452 and 608 nm). Irradiation of the solution with 599 nm light resulted in a bathochromic shift of the absorbance (*λ*_max_ = 461 and 655 nm), characteristic of hemi‐indigo *Z*→*E* isomerization.^[^ ^43^, ^44^, ^45^, ^46^, ^53^ ^]^ Interestingly, when subsequently irradiating the sample with 455 nm light, around the maximum where the *E*‐isomer has higher absorption than the *Z*‐isomer, the spectrum almost did not change, indicating that a similar photostationary state (PSS) ratio was attained. During irradiation, a clear isosbestic point was maintained at *λ* = 638 nm (Figure S11). Also, when subsequently irradiating close to this isosbestic point with 637 nm light, only minor spectral changes were observed. Hence, these UV–vis studies indicate that similar PSS ratios are obtained at the used irradiation wavelengths (i.e., 599, 455, and 637 nm).^[^ ^54^ ^]^ Further, the spectrum recorded at PSS only minimally changed over the course of 1 h in the dark (Figure S12), indicating high thermal stability.

**Figure 2:**
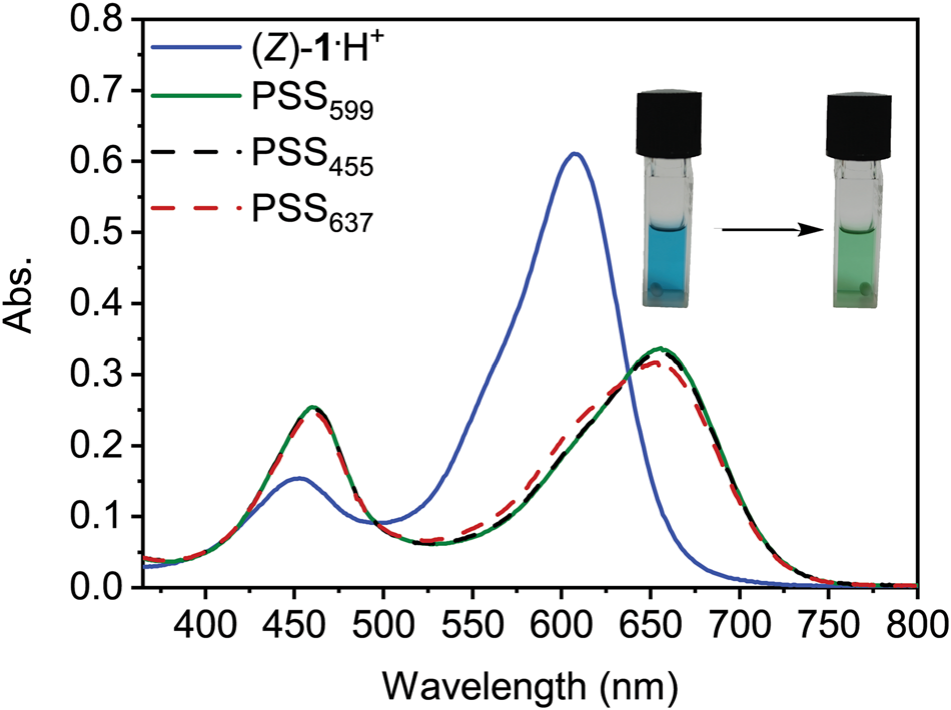
UV–vis spectral changes of (*Z*)‐**1** ^.^H^+^ (10 µm in MeOH, 12 equiv. TFA, 293 K) upon sequential irradiation with 599, 455, and 637 nm light.

Next, this photoisomerization process was followed by ^1^H NMR spectroscopy to quantify the *E*/*Z* ratios at the PSS. Upon 599 nm irradiation of (*Z*)‐**1** ^.^H^+^ in MeOD (containing 10 equiv. of TFA to ensure full protonation), a new set of signals appeared, while the original signals nearly disappeared (Figures S15–S17). The new signal set was assigned to the *E*‐isomer, and by relative signal integration, the PSS_599_ *E*/*Z*‐ratio was determined as 95:5. When the sample was subsequently irradiated with 455 and 637 nm light, only minor spectral changes were observed, and the PSS *E*/*Z*‐ratios were found to be 91:9 and 86:14, respectively. This result confirms that, at the three different irradiation wavelengths used, the photoequilibrium lies toward the *E*‐isomer. A similar observation was made previously for other heterocyclic hemi‐(thio)indigo photoswitches able to form an intramolecular hydrogen bond. This high photoconversion has been ascribed to a reduction in quantum yield for isomerization from the intramolecularly hydrogen‐bonded isomer.^[^ ^43^, ^44^, ^45^, ^46^ ^]^

Then, binding of chloride was studied by ^1^H NMR titrations in CD_2_Cl_2_/MeOD (95:5 v/v, 4 equiv. of TFA).^[^ ^55^ ^]^ For (*Z*)‐**1** ^.^H^+^, addition of NBu_4_Cl caused significant changes in chemical shifts, in particular of protons located close to the binding site, as a sign of chloride complexation (Figure S18). These chemical shift changes were fitted to a 1:1 binding model with HypNMR,^[^ ^56^ ^]^ affording an association constant of *K*_a_ = (2.4 ± 0.7) × 10^4^ m ^−1^ (Figure S19). A similar titration for the *E*‐isomer, which was prepared by 599 nm irradiation of a solution of the *Z*‐isomer, gave much smaller chemical shift changes (Figure S20). While these smaller changes could not be accurately fitted to a 1:1 binding model, they are an indication of weaker binding of chloride to the *E*‐isomer compared to the *Z*‐isomer.

Subsequently, chloride transport activity and selectivity were assessed using a cationophore‐coupled ion‐selective electrode (ISE) assay.^[^ ^57^ ^]^ For this assay, POPC liposomes are prepared with an internal solution of KCl (300 mm) and an external solution of potassium gluconate (KGlu) (300 mm), both buffered to the desired pH value. The established chloride concentration gradient can only be dissipated by an anion transporter when coupled with an appropriate cationophore; otherwise, buildup of charge or formation of a pH gradient will impede transport. The natural product valinomycin acts as an electrogenic potassium carrier and couples with an electrogenic chloride transporter to facilitate net KCl efflux. Conversely, monensin operates strictly through electroneutral K^+^/H^+^ exchange and couples with an electroneutral HCl transporter to give net KCl efflux. Comparing transport activity in the presence of either cationophore therefore provides insight into the preferred mechanism (i.e., electrogenic Cl^−^ or electroneutral HCl transport; see Figure S23 for a schematic explanation).

The ISE assay was first performed at pH 7.2 with (*Z*)‐**1** ^.^H^+^ added to the liposome solution from MeOH (0.2 mol% with respect to POPC lipids). In the presence of valinomycin (0.1 mol%), negligible chloride efflux was observed, while in the presence of monensin (0.1 mol%), chloride transport was significant (Figure S24). This difference confirms the anticipated selectivity for the electroneutral HCl transport mechanism. That is, chloride is bound and transported across the bilayer by the protonated dipyrrin‐derived hemi‐indigo in a charge‐neutral complex, while the compound diffuses back to the inner leaflet in deprotonated form.^[^ ^24^ ^]^ In addition, a dose‐response curve was constructed in the presence of monensin. By fitting the transport data to the Hill equation, the half‐maximal effective concentration was determined as EC_50_ = 0.076 mol% (see Table 1; Figures 3b and S25), showing that the *Z*‐isomer is a highly active transporter.

**Table 1:** Comparison of EC_50_ values of (*Z*)‐**1** and (*E*_PSS_)‐**1** at pH 7.2 and 5.5.

pH	EC_50_ (mol%)^a)^ (*Z*)‐1	EC_50_ (mol%)^a)^ (*E*_PSS_)‐1	*F*_(_ *_E__/__Z_*_)_ ^b)^
7.2	(7.57 ± 0.8) × 10^−2^	1.18 ± 0.1	15.6
5.5	(1.10 ± 0.1) × 10^−2^	(1.53 ± 0.2) × 10^−1^	13.9

^a)^ EC_50_ is defined as the concentration (in mol% with respect to POPC lipids) required to reach 50% of the maximum activity at *t* = 360 s in the ISE assay as obtained by Hill analysis.

^b)^ Factor decrease in transport activity by irradiation of (*Z*)‐**1** to (*E*_PSS_)‐**1** [*F* = EC_50(_ *_E_*_)_/EC_50(_ *_Z_*_)_].

**Figure 3:**
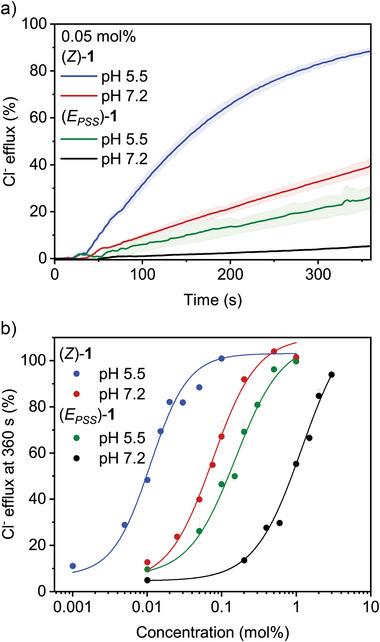
a) Chloride efflux over time mediated by (*Z*)‐**1** or (*E*_PSS_)‐**1** (0.05 mol% with respect to lipids) in the presence of monensin at pH 7.2 and 5.5. b) Hill plots of chloride efflux as a function of concentration for (*Z*)‐**1** and photogenerated (*E*_PSS_)‐**1** at pH 7.2 and 5.5.

When the same assay with (*Z*)‐**1** ^.^H^+^ was performed at pH 5.5, higher transport activity was observed than at pH 7.2 (Figure 3a), whereas the strict selectivity for electroneutral HCl transport was retained (Figure S26). Hill analysis now gave a half‐maximal effective concentration of EC_50_ = 0.011 mol% (Figures 3b and S27), which is seven times lower than the value determined at pH 7.2. The higher transport activity at a lower pH value supports that the protonated form of (*Z*)‐**1** is the active chloride carrier, mechanistically similar to prodigiosin.^[^ ^11^, ^12^, ^13^ ^]^

As no suitable chloride binding pocket is available in the *E*‐isomer, we expected deactivation of transport by light irradiation. Therefore, the transport activity of the (*E*_PSS_)‐mixture, obtained by irradiating a solution of (*Z*)‐**1^.^** H^+^ in MeOH with 599 nm light, was studied using the same ISE assay. To our delight, chloride efflux with (*E*_PSS_)‐**1** was much lower than with (*Z*)‐**1** at both pH values (Figure 3a). Hill analysis revealed a 16‐fold and 14‐fold decrease in HCl transport activity at pH 7.2 and 5.5, respectively (Table 1; Figures 3b and S28–S33). The residual activity and pH sensitivity observed for (*E*_PSS_)‐**1** is partially attributed to the remaining *Z*‐isomer.

To gain insight into the observed pH dependence of transport activity, the UV–vis absorbance of both isomers of **1** in the POPC liposome at pH 5.5 and 7.2 was compared to that in MeOH solution in the presence of TFA and Et_3_N (Figures S36–S39). In MeOH, the absorption of the protonated species is red‐shifted with respect to that of the neutral species. Interestingly, the spectra recorded for the liposome samples at pH 5.5 and 7.2 closely resembled those taken in MeOH in the presence of TFA and Et_3_N, respectively. Additionally, various UV–vis spectra were recorded for liposomes containing the *Z*‐isomer in the pH range between 4 and 8, showing an inflection point around pH 6.7 (Figure S40). These results confirm that the transporter is in a protonated state at pH 5.5 and predominantly deprotonated at pH 7.2. The observed increase in transport activity at lower pH thus originates from facilitated protonation of the transporter.

Finally, we monitored the effect of in situ irradiation after incorporation of the compound into the bilayer and prior to the addition of monensin (which initiates transport). Gratifyingly, the irradiated liposome samples showed strongly diminished chloride efflux both at pH 5.5 and 7.2 (Figures 4, S34, and S35). In a separate experiment, UV–vis spectra of (*Z*)‐**1** in the POPC bilayer were recorded before and after 599 nm irradiation to confirm that this decrease in transport activity is due to photoinduced *Z*→*E* isomerization. Indeed, irradiation resulted in a red shift of the maximum and an overall decrease of the absorption at both pH 5.5 and 7.2 (Figures S41 and S42), reminiscent of what was observed in MeOH solution in the presence of TFA and Et_3_N (Figures S11 and S13). Importantly, the deactivating effect of in situ irradiation verifies that the distinct activity of the two isomers is not due to an intrinsic difference in membrane deliverability.^[^ ^37^ ^]^

**Figure 4:**
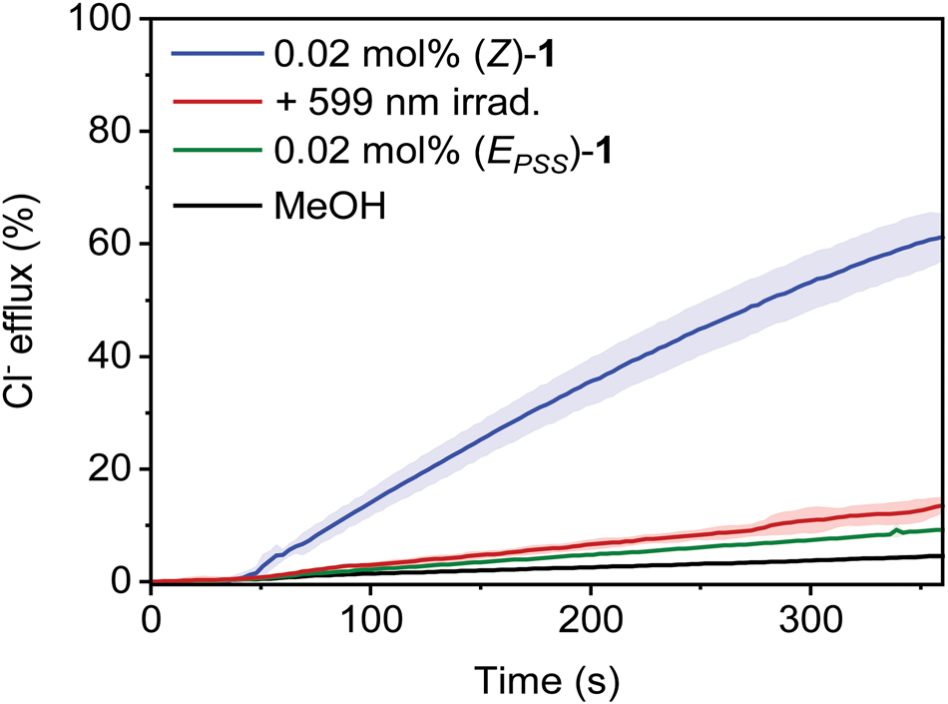
Chloride efflux over time measured for (*Z*)‐**1** added from MeOH (0.02 mol% with respect to lipids) with and without 599 nm irradiation of the liposome sample prior to addition of monensin. The traces are compared to that of (*E*_PSS_)‐**1** pregenerated in and added from MeOH solution (0.02 mol% with respect to lipids) and an experiment in which only MeOH was added.

In summary, we have developed a pH‐ and red‐light‐responsive prodigiosin‐like transporter by dipyrrin functionalization of hemi‐indigo. The *Z*‐isomer strongly binds chloride when protonated and facilitates strictly electroneutral HCl symport across the POPC bilayer membrane. Importantly, it is more active in an acidic environment (at pH 5.5 compared to pH 7.2) and can be deactivated by visible‐light irradiation. That is, irradiation gives high conversion to the intramolecularly hydrogen‐bonded, less active *E*‐isomer. Such efficient deactivation of transport by visible light is unprecedented and will be useful to reduce undesired cytotoxicity in healthy tissue and/or after excretion into the environment.

## Supporting Information

The authors have cited additional references within the Supporting Information.^[^ ^58^, ^59^, ^60^, ^61^ ^]^

## Conflict of Interests

The authors declare no conflict of interest.

## Supporting information



Supporting Information

## Data Availability

The data that support the findings of this study are available in the Supporting Information of this article.

## References

[1] J. T. Davis , P. A. Gale , R. Quesada , Chem. Soc. Rev. 2020, 49, 6056–6086, 10.1039/C9CS00662A. 32692794

[2] A. Mondal , M. Ahmad , D. Mondal , P. Talukdar , Chem. Commun. 2023, 59, 1917–1938, 10.1039/D2CC06761G. 36691926

[3] E. Feo , P. A. Gale , Curr. Opin. Chem. Biol. 2024, 83, 102535, 10.1016/j.cbpa.2024.102535. 39341172

[4] H. Li , J. J. Salomon , D. N. Sheppard , M. A. Mall , L. J. V. Galietta , Curr. Opin. Pharmacol. 2017, 34, 91–97, 10.1016/j.coph.2017.10.002. 29065356

[5] H. Li , H. Valkenier , A. G. Thorne , C. M. Dias , J. A. Cooper , M. Kieffer , N. Busschaert , P. A. Gale , D. N. Sheppard , A. P. Davis , Chem. Sci. 2019, 10, 9663–9672, 10.1039/C9SC04242C. 32055336 PMC6984391

[6] R. Quesada , R. Dutzler , J. Cystic Fibrosis 2020, 19, S37–S41, 10.1016/j.jcf.2019.10.020. 31662238

[7] S.‐K. Ko , S. K. Kim , S. Share , V. M. Lynch , J. Park , W. Namkung , W. Van Rossom , N. Busschaert , P. A. Gale , J. L. Sessler , I. Shin , Nat. Chem. 2014, 6, 885–892, 10.1038/nchem.2021. 25242483

[8] E. Hernando , V. Soto‐Cerrato , S. Cortés‐Arroyo , R. Pérez‐Tomás , R. Quesada , Org. Biomol. Chem. 2014, 12, 1771–1778, 10.1039/C3OB42341G. 24500335

[9] S.‐H. Park , S.‐H. Park , E. N. W. Howe , J. Y. Hyun , L.‐J. Chen , I. Hwang , G. Vargas‐Zuñiga , N. Busschaert , P. A. Gale , J. L. Sessler , I. Shin , Chem 2019, 5, 2079–2098, 10.1016/j.chempr.2019.05.001. 33791443 PMC8009298

[10] L. E. Brennan , L. K. Kumawat , M. E. Piatek , A. J. Kinross , D. A. McNaughton , L. Marchetti , C. Geraghty , C. Wynne , H. Tong , O. N. Kavanagh , F. O'Sullivan , C. S. Hawes , P. A. Gale , K. Kavanagh , R. B. P. Elmes , Chem 2023, 9, 3138–3158, 10.1016/j.chempr.2023.07.014.

[11] T. Sato , H. Konno , Y. Tanaka , T. Kataoka , K. Nagai , H. H. Wasserman , S. Ohkuma , J. Biol. Chem. 1998, 273, 21455–21462, 10.1074/jbc.273.34.21455. 9705273

[12] J. L. Sessler , L. R. Eller , W.‐S. Cho , S. Nicolaou , A. Aguilar , J. T. Lee , V. M. Lynch , D. J. Magda , Angew. Chem. Int. Ed. 2005, 44, 5989–5992, 10.1002/anie.200501740. 16114075

[13] J. L. Seganish , J. T. Davis , Chem. Commun. 2005, 5781–5783, 10.1039/b511847f. 16307144

[14] B. Díaz de Greñu , P. I. Hernández , M. Espona , D. Quiñonero , M. E. Light , T. Torroba , R. Pérez‐Tomás , R. Quesada , Chem.‐Eur. J. 2011, 17, 14074–14083, 10.1002/chem.201101547. 22069220

[15] W. Van Rossom , D. J. Asby , A. Tavassoli , P. A. Gale , Org. Biomol. Chem. 2016, 14, 2645–2650, 10.1039/C6OB00002A. 26905059

[16] L. A. Jowett , E. N. W. Howe , V. Soto‐Cerrato , W. Van Rossom , R. Pérez‐Tomás , P. A. Gale , Sci. Rep. 2017, 7, 9397, 10.1038/s41598-017-09645-9. 28839192 PMC5570892

[17] N. Busschaert , R. B. P. Elmes , D. D. Czech , X. Wu , I. L. Kirby , E. M. Peck , K. D. Hendzel , S. K. Shaw , B. Chan , B. D. Smith , K. A. Jolliffe , P. A. Gale , Chem. Sci. 2014, 5, 3617–3626, 10.1039/C4SC01629G. 26146535 PMC4486358

[18] R. B. P. Elmes , N. Busschaert , D. D. Czech , P. A. Gale , K. A. Jolliffe , Chem. Commun. 2015, 51, 10107–10110, 10.1039/C5CC03625A. 25998008

[19] S. V. Shinde , P. Talukdar , Angew. Chem. Int. Ed. 2017, 56, 4238–4242, 10.1002/anie.201700803. 28276656

[20] A. Saha , N. Akhtar , V. Kumar , S. Kumar , H. K. Srivastava , S. Kumar , D. Manna , Org. Biomol. Chem. 2019, 17, 5779–5788, 10.1039/C9OB00650H. 31135015

[21] L. Tapia , Y. Pérez , M. Bolte , J. Casas , J. Solá , R. Quesada , I. Alfonso , Angew. Chem. Int. Ed. 2019, 58, 12465–12468, 10.1002/anie.201905965. 31298461

[22] S. V. Shinde , P. Talukdar , Org. Biomol. Chem. 2019, 17, 4483–4490, 10.1039/C9OB00554D. 30984952

[23] P. V. Santacroce , J. T. Davis , M. E. Light , P. A. Gale , J. C. Iglesias‐Sánchez , P. Prados , R. Quesada , J. Am. Chem. Soc. 2007, 129, 1886–1887, 10.1021/ja068067v. 17253691

[24] E. N. W. Howe , N. Busschaert , X. Wu , S. N. Berry , J. Ho , M. E. Light , D. D. Czech , H. A. Klein , J. A. Kitchen , P. A. Gale , J. Am. Chem. Soc. 2016, 138, 8301–8308, 10.1021/jacs.6b04656. 27299473

[25] A. Castellano‐Hinojosa , M. J. Gallardo‐Altamirano , J. González‐López , A. González‐Martínez , J. Hazard. Mater. 2023, 447, 130818, 10.1016/j.jhazmat.2023.130818. 36680899

[26] W. A. Velema , W. Szymanski , B. L. Feringa , J. Am. Chem. Soc. 2014, 136, 2178–2191, 10.1021/ja413063e. 24456115

[27] K. Hüll , J. Morstein , D. Trauner , Chem. Rev. 2018, 118, 10710–10747, 10.1021/acs.chemrev.8b00037. 29985590

[28] W. Lee , Z.‐H. Li , S. Vakulenko , S. Mobashery , J. Med. Chem. 2000, 43, 128–132, 10.1021/jm980648a. 10633044

[29] W. A. Velema , J. P. van der Berg , M. J. Hansen , W. Szymanski , A. J. M. Driessen , B. L. Feringa , Nat. Chem. 2013, 5, 924–928, 10.1038/nchem.1750. 24153369

[30] M. Borowiak , W. Nahaboo , M. Reynders , K. Nekolla , P. Jalinot , J. Hasserodt , M. Rehberg , M. Delattre , S. Zahler , A. Vollmar , D. Trauner , O. Thorn‐Seshold , Cell 2015, 162, 403–411, 10.1016/j.cell.2015.06.049. 26165941

[31] J. de Jong , J. E. Bos , S. J. Wezenberg , Chem. Rev. 2023, 123, 8530–8574, 10.1021/acs.chemrev.3c00039. 37342028 PMC10347431

[32] M. Ahmad , S. A. Gartland , M. J. Langton , Angew. Chem. Int. Ed. 2023, 62, e202308842, 10.1002/anie.202308842. 37478126

[33] Y. R. Choi , G. C. Kim , H.‐G. Jeon , J. Park , W. Namkung , K.‐S. Jeong , Chem. Commun. 2014, 50, 15305–15308, 10.1039/C4CC07560A. 25350406

[34] A. Kerckhoffs , M. J. Langton , Chem. Sci. 2020, 11, 6325–6331, 10.1039/D0SC02745F. 32953027 PMC7472928

[35] M. Ahmad , S. Chattopadhayay , D. Mondal , T. Vijayakanth , P. Talukdar , Org. Lett. 2021, 23, 7319–7324, 10.1021/acs.orglett.1c02249. 34519509

[36] S. J. Wezenberg , L. J. Chen , J. E. Bos , B. L. Feringa , E. N. W. Howe , X. Wu , M. A. Siegler , P. A. Gale , J. Am. Chem. Soc. 2022, 144, 331–338, 10.1021/jacs.1c10034. 34932344 PMC8759083

[37] J. E. Bos , M. A. Siegler , S. J. Wezenberg , J. Am. Chem. Soc. 2024, 146, 31085–31093, 10.1021/jacs.4c10952. 39485737 PMC11565646

[38] P. Ferreira , G. Aragay , P. Ballester , Org. Chem. Front. 2024, 11, 2468–2476, 10.1039/D4QO00399C.

[39] Z. Kokan , M. J. Chmielewski , J. Am. Chem. Soc. 2018, 140, 16010–16014, 10.1021/jacs.8b08689. 30415543

[40] J. de Jong , B. L. Feringa , S. J. Wezenberg , ChemPhysChem 2019, 20, 3306–3310, 10.1002/cphc.201900917. 31622003 PMC6972635

[41] B. Shao , H. Fu , I. Aprahamian , Science 2024, 385, 544–549, 10.1126/science.adp3506. 39088617

[42] M. Vega , L. Martínez‐Crespo , M. Barceló‐Oliver , C. Rotger , A. Costa , Org. Lett. 2023, 25, 3423–3428, 10.1021/acs.orglett.3c00993. 37158572 PMC10204084

[43] M. Ikegami , T. Arai , Bull. Chem. Soc. Jpn. 2003, 76, 1783–1792, 10.1246/bcsj.76.1783.

[44] V. Josef , F. Hampel , H. Dube , Angew. Chem. Int. Ed. 2022, 61, e202210855, 10.1002/anie.202210855. PMC9826360 36040861

[45] M. Krell‐Jørgensen , H. Zulfikri , M. G. Bonnevie , F. S. Bro , A. O. Dohn , L. Laraia , Chem. Commun. 2023, 59, 563–566, 10.1039/D2CC05548A. 36537010

[46] N. Duindam , M. van Dongen , M. A. Siegler , S. J. Wezenberg , J. Am. Chem. Soc. 2023, 145, 21020–21026, 10.1021/jacs.3c06587. 37712835 PMC10540201

[47] T. Arai , M. Ikegami , Chem. Lett. 1999, 28, 965–966, 10.1246/cl.1999.965.

[48] T. E. Wood , A. Thompson , Chem. Rev. 2007, 107, 1831–1861, 10.1021/cr050052c. 17430001

[49] J. A. van Koeveringe , J. Lugtenburg , Recl. Trav. Chim. Pays‐Bas 1977, 96, 55–57, 10.1002/recl.19770960209.

[50] C. Wan , A. Burghart , J. Chen , F. Bergström , L. B.‐Å. Johansson , M. F. Wolford , T. G. Kim , M. R. Topp , R. M. Hochstrasser , K. Burgess , Chem.‐Eur. J. 2003, 9, 4430–4441, 10.1002/chem.200304754. 14502630

[51] C. Uriel , D. Grenier , F. Herranz , N. Casado , J. Bañuelos , E. Rebollar , I. Garcia‐Moreno , A. M. Gomez , J. C. López , J. Org. Chem. 2024, 89, 4042–4055, 10.1021/acs.joc.3c02907. 38438277 PMC10949249

[52] Photoisomerization of the neutral species was also studied, but spectral complexity is introduced by presumed tautomeric equilibria; see Figures S13‐S14 for further information.

[53] C. Petermayer , H. Dube , Acc. Chem. Res. 2018, 51, 1153–1163, 10.1021/acs.accounts.7b00638. 29694014

[54] Selective irradiation of the *E*‐isomer using a high‐power LED emitting around 730 nm resulted in very slow isomerization back to the *Z*‐isomer as was noted by minor changes in the UV‐Vis spectrum after 1 h.

[55] Negligible binding was observed in MeOD (with 2 equiv. TFA); see Figures S21‐S22.

[56] C. Frassineti , S. Ghelli , P. Gans , A. Sabatini , M. S. Moruzzi , A. Vacca , Anal. Biochem. 1995, 231, 374–382, 10.1006/abio.1995.9984. 8594988

[57] X. Wu , E. N. W. Howe , P. A. Gale , Acc. Chem. Res. 2018, 51, 1870–1879, 10.1021/acs.accounts.8b00264. 30063324

[58] M. J. Frisch , G. W. Trucks , H. B. Schlegel , G. E. Scuseria , M. A. Robb , J. R. Cheeseman , G. Scalmani , V. Barone , G. A. Petersson , H. Nakatsuji , X. Li , M. Caricato , A. V. Marenich , J. Bloino , B. G. Janesko , R. Gomperts , B. Mennucci , H. P. Hratchian , J. V. Ortiz , A. F. Izmaylov , J. L. Sonnenberg , D. Williams‐Young , F. Ding , F. Lipparini , F. Egidi , J. Goings , B. Peng , A. Petrone , T. Henderson , D. Ranasinghe , et al., Gaussian 16, Revision C.01, Gaussian, Inc., Wallingford, CT 2016.

[59] Avogadro: an open‐source molecular builder and visualization tool. Version 1.2.0 http://avogadro.cc/.

[60] R. Dennington , T. A. Keith , J. M. Millam , GaussView, Version 6, Semichem Inc., Shawnee Mission, KS 2016.

[61] P. Pracht , F. Bohle , S. Grimme , Phys. Chem. Chem. Phys. 2020, 22, 7169–7192, 10.1039/C9CP06869D. 32073075

