# Supplementary material for: Hemi‐Indigiosin: A pH and Red‐Light Responsive Transmembrane HCl Transporter
Source: Angew Chem Int Ed Engl. 2025 Oct 30;64(51):e15930. doi: 10.1002/anie.202515930 (PMC12707358; doi:10.1002/anie.202515930)
Supplement: Supplementary file 1 — Supporting Information [file ANIE-64-e15930-s001.pdf]

SUPPORTING INFORMATION

Hemi-indigiosin: A pH and Red-Light Responsive Transmembrane HCl Transporter

Nol Duindam,[a] Jasper E. Bos[a], Felix van Nifterik,[a] and Sander J. Wezenberg[a]*

[a] *Leiden Institute of Chemistry, Leiden University, Einsteinweg 55, 2333 CC Leiden, The Netherlands*

Email: s.j.wezenberg@lic.leidenuniv.nl

Table of Contents

1. Experimental section S2

2. ^{1}H , ^{19}F and ^{13}C NMR spectra of (*Z*)-**1** S6

3. UV-Vis photoisomerization studies S15

4. ^{1}H NMR photoisomerization studies S18

5. ^{1}H NMR titration studies S20

6. Cationophore-coupled ISE assays S25

7. UV-Vis studies in POPC liposomes S39

8. DFT Calculations S43

9. References S50

1. Experimental section

General methods and materials

Pyrrole-derived hemi-indigo (*Z*)-**S1** was synthesized according to a procedure reported in the literature.[1] All other chemicals were commercially available and were used without further purification. Flash chromatography (FC) was performed using SiO_2 (60 M, 0.04-0.063 mm) or C_{18}-functionalized SiO_2 (5.4g, high-efficiency spherical C18, 15um, 100A,C 17%, end-capped, 320m^2/g) purchased from Screening Devices BV. Thin-layer chromatography (TLC) was carried out on aluminum sheets coated with silica (60 Å, UV254 indicator) or coated with C_{18}-functionalized SiO_2 (0.15 mm C_{18} silica gel with UV254 indicator, Macherey Nagel Alugram Plates). Compounds were visualized with UV light (254 nm or 365 nm). ^{1}H, ^{19}F, and ^{13}C NMR spectra were recorded on Bruker AV 400 MHz, Bruker 500 MHz Ultra Shield, Bruker AV-III 600 MHz, and Bruker AV-III-HD 850 MHz instruments at 298 K unless indicated otherwise. Chemical shifts (δ) are denoted in parts per million (ppm) relative to residual protiated solvent (CD_2Cl_2: for ^{1}H detection, δ = 5.32 ppm; for ^{13}C detection, δ = 53.84 ppm, MeOD: for ^{1}H detection, δ = 3.31 ppm; for ^{13}C detection, δ = 49.00 ppm). The splitting pattern of peaks is designated as follows: s (singlet), d (doublet), t (triplet), q (quartet), p (quintet), h (septet), m (multiplet), br (broad). High-resolution mass spectrometry (ESI-MS) was performed on a Thermo Scientific Q Exactive HF spectrometer with ESI ionization. IR spectra were recorded on a Perkin Elmer Spectrum Two FT-IR spectrometer. The intensity of bands (ν = cm^{-1}) is assigned as follows: s (strong), m (medium), w (weak), very w (very weak), br (broad), and sh (shoulder). Melting points were determined with a Büchi M560 apparatus. UV-Vis spectra were recorded on an Agilent Cary 8454 spectrometer or a Perkin Elmer Lambda 465 spectrometer using 1 cm or 1 mm quartz cuvettes. Irradiation of UV-Vis and NMR samples was carried out using LEDs purchased from Thorlabs: M455F3 (17 mW minimal LED output, 1000 mA, peak wavelength = 455 nm, FWHM = 14 nm), M590L4 (230 mW minimal LED output power, 1000 mA, peak wavelength = 599 nm, FWHM = 15 nm), M625L4 (700 mW minimal LED output power, 1000 mA, peak wavelength = 637 nm, FWHM = 17 nm). In this work, the peak wavelength (maximum emission intensity) is referred to, rather than following convention by Thorlabs of taking the nominal wavelength (defined as the wavelength at which the LED appears brightest to the human eye).

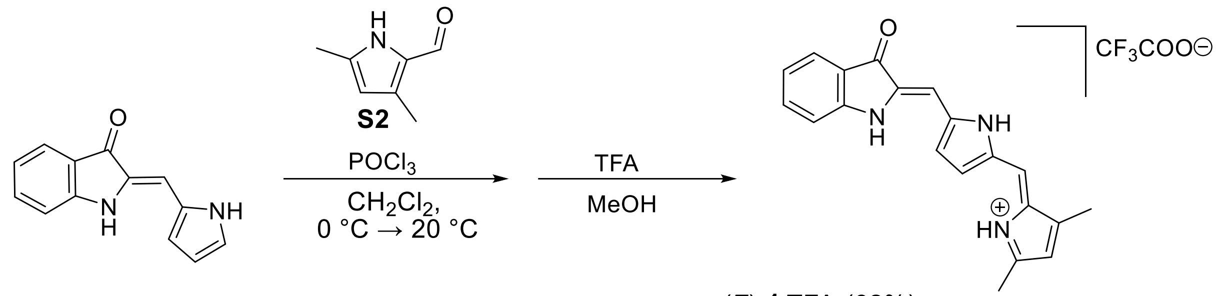

Scheme S1. Synthetic route toward hemi-indigo (*Z*)-**1**·TFA.

General procedure for the MacDonald-type condensation:

The reagent to mediate the condensation (see Table S1) was added to 3,5-Dimethyl-1*H*-pyrrole-2-carbaldehyde (**S2**) and (*Z*)-**S1** (20 mg, 0.095 mmol) in dry and degassed MeOH (4 mL) under argon atmosphere. After the indicated reaction time, the reaction mixture was concentrated *in vacuo* and the conversion was determined by ^{1}H NMR spectroscopy. In case of significant conversion, the desired product was purified by FC (C_{18} SiO_2; CH_3CN:H_2O 2:8 to 8:2 with 0.1 v% TFA) and freeze-dried to obtain the TFA salt. ^{1}H NMR spectral characterization matched that of the product obtained by the improved method as described below.

Table S1. Used conditions to mediate the condensation reaction of (*Z*)-**S1** with aldehyde **S2**.

Equiv. S2	Reagent (equiv.)	Solvent	Temp.	Time	% (*Z*)-1
1.0	HBr[a] (5.0)	MeOH	20 °C	12 h	trace
1.0	*p*TsOH (5.0)	MeOH	20 °C	30 min	trace
1.6	HCl[b] (10.0)	MeOH	20 °C	3 h	2%[d]
0.5	HCl[b] (10.0)	MeOH	20 °C	3 h	2%[d]
1.6	TFA (5.0)	MeOH	20 °C	3 h	4%[d]
1.6[c]	TFA (5.0)	MeOH	20 °C	3 h	3%[d]
1.6	$InCl_3$ (0.2)	MeOH	20 °C	48 h	None

[a] Added as a 33 wt% solution in acetic acid. [b] Added as a 0.5 M solution in MeOH. [c] **S2** was added as a MeOH solution (2 mL) over a period of 1 h to a premixed solution of (*Z*)-**S1** and TFA (2 mL MeOH). [d] Isolated yields (after purification by FC) are provided.

Dipyrrin-derived hemi-indigo (*Z*)-1·TFA

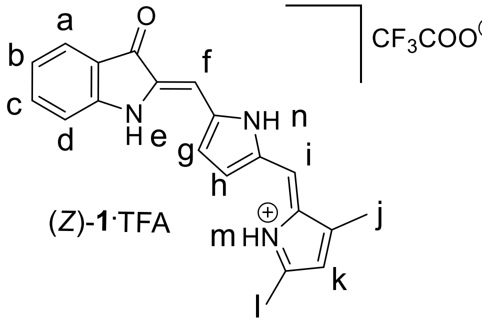

3,5-Dimethyl-1*H*-pyrrole-2-carbaldehyde (21 mg, 0.17 mmol) and (*Z*)-**S1** (30 mg, 0.14 mmol) were dissolved in dried and degassed CH_2Cl_2 (3 mL) under argon atmosphere. The stirring solution was cooled to 0 °C and $POCl_3$ (36 µL, 0.43 mmol) was added dropwise over a period of 1 minute. The mixture was stirred at 0 °C for an additional 5 minutes, after which it was allowed to warm to rt and stirred for a further 15 minutes. The mixture was then treated with MeOH (1 mL) and poured into petroleum ether (60 mL) and, while cooled at 4 °C, a fine precipitate formed after 30 minutes. The supernatant was removed and the precipitate was redissolved in MeOH (1 mL) containing TFA (20 µL). Addition to petroleum ether (60 mL) again led to precipitation and after removal of the supernatant, the precipitate was dissolved in $CHCl_3$ (5 mL) and precipitated by addition of petroleum ether (10 mL, performed twice). The precipitate was then dispersed in MilliQ water containing TFA (0.1 v%) and lyophilized to afford the TFA salt of (*Z*)-**1** as a dark purple solid (38 mg, 62%).

Note: In the ^{13}C NMR spectrum acquired at 298 K in MeOD three carbon signals are missing, *i.e.* a quaternary carbon signal and two pyrrolic carbon signals. These signals most likely broaden into the baseline due to rotamer interconversion. Therefore, ^{1}H and ^{13}C NMR spectra were additionally recorded in CD_2Cl_2 in presence of NBu_4Cl (2 equiv.) and TFA (2 equiv.) at 263 K. Under these conditions the rotamer-induced peak broadening is reduced, and all ^{13}C signals are observed.

R_f = 0.12 (SiO_2-C_{18}; CH_3CN/H_2O 1:1 with 0.1 v% TFA); m.p. > 300 °C decomp; ^{1}H NMR (850 MHz, MeOD, assignment based on 2D COSY and NOESY spectra) δ = 7.77 (s, 1H; H**h**), 7.65 (d, J = 7.5 Hz, 1H; H**a**), 7.58 (s, 1H; H**i**), 7.56 (ddd, J = 8.3, 7.2, 1.3 Hz, 1H; H**c**), 7.36 (d, J = 4.6 Hz, 1H; H**g**), 7.16 (d, J = 8.0 Hz, 1H; H**d**), 7.02 (td, J = 7.4, 0.8 Hz, 1H; H**b**), 6.62 (s, 1H; H**f**), 6.51 (s, 1H; H**k**), 2.59 (s, 3H; H**l**), 2.42 (s, 3H; H**j**); ^{1}H NMR (600 MHz, CD_2Cl_2, 2 equiv. TFA and 2 equiv. NBu_4Cl, 263 K) δ 14.09 (s, 1H; H**m**), 12.72 (s, 1H; H**n**), 11.07 (s, 1H; H**e**),

7.60 (d, $J = 7.5$ Hz, 1H; H**a**), 7.43 (t, $J = 7.6$ Hz, 1H; H**c**), 7.24 (d, $J = 8.0$ Hz, 1H; H**d**), 7.15 (s, 1H; H**h**), 7.01 (s, 1H; H**i**), 6.96 – 6.91 (m, 2H; H**b** and H**g**), 6.55 (s, 1H; H**f**), 6.28 (s, 1H; H**k**), 2.66 (s, 3H; H**l**), 2.32 (s, 3H; H**j**); ^{13}C NMR (214 MHz, MeOD) $\delta =$ 188.1, 161.9, 154.8, 151.7, 138.8, 138.2, 133.2, 132.2, 125.7, 125.0, 122.4, 121.4, 121.0, 113.6, 96.8, 14.7, 12.1; ^{13}C NMR (151 MHz, CD_2Cl_2, 2 equiv. NBu_4Cl and TFA, 263 K, assignment based on 2D COSY and NOESY spectra) δ 187.1, 159.6, 153.4, 149.0, 147.1, 136.6, 136.1, 134.8, 131.3, 129.8, 124.7, 122.2, 121.5, 121.1, 120.2, 119.4, 112.9, 96.1, 14.9, 12.4; ^{19}F NMR (376 MHz, MeOD) δ –76.6; IR (ATR) ν = 3225 (br, w), 3128 (w), 3001 (very w), 1687 (m, sh), 1671 (m), 1614 (s), 1560 (s, sh), 1543 (m), 1517.8 (w), 1482 (w), 1466 (w, sh), 1424 (very w), 1383 (m), 1324 (very w), 1313 (very w), 1285 (m), 1265 (w), 1246 (very w), 1193 (s), 1176 (m), 1127 (s), 1076 (m), 964 (m), 954 (m, sh), 941 (m, sh), 890 (very w), 833 (m), 800 (m), 778 (w), 756 (m), 720 (m), 710 (w, sh), 655 (m), 617 (w); UV-Vis (MeOH): λ_{max} (ε) = 452 nm (1.5×10^4 M^{-1} cm^{-1}), 608 nm (6.1×10^4 M^{-1} cm^{-1}); HRMS (ESI) *m/z:* 316.1444 ($[M+H]^+$, calcd for $C_{20}H_{18}N_3O^+$: 316.1444); elemental analysis calcd (%) for [$C_{20}H_{18}N_3O^+$ + CF_3COO^-]: 61.54 %, H: 4.23%, N: 9.79%. C: 61.02 %, H: 4.44%, N: 9.63%.

2. ^{1}H , ^{19}F and ^{13}C NMR spectra of (*Z*)-1

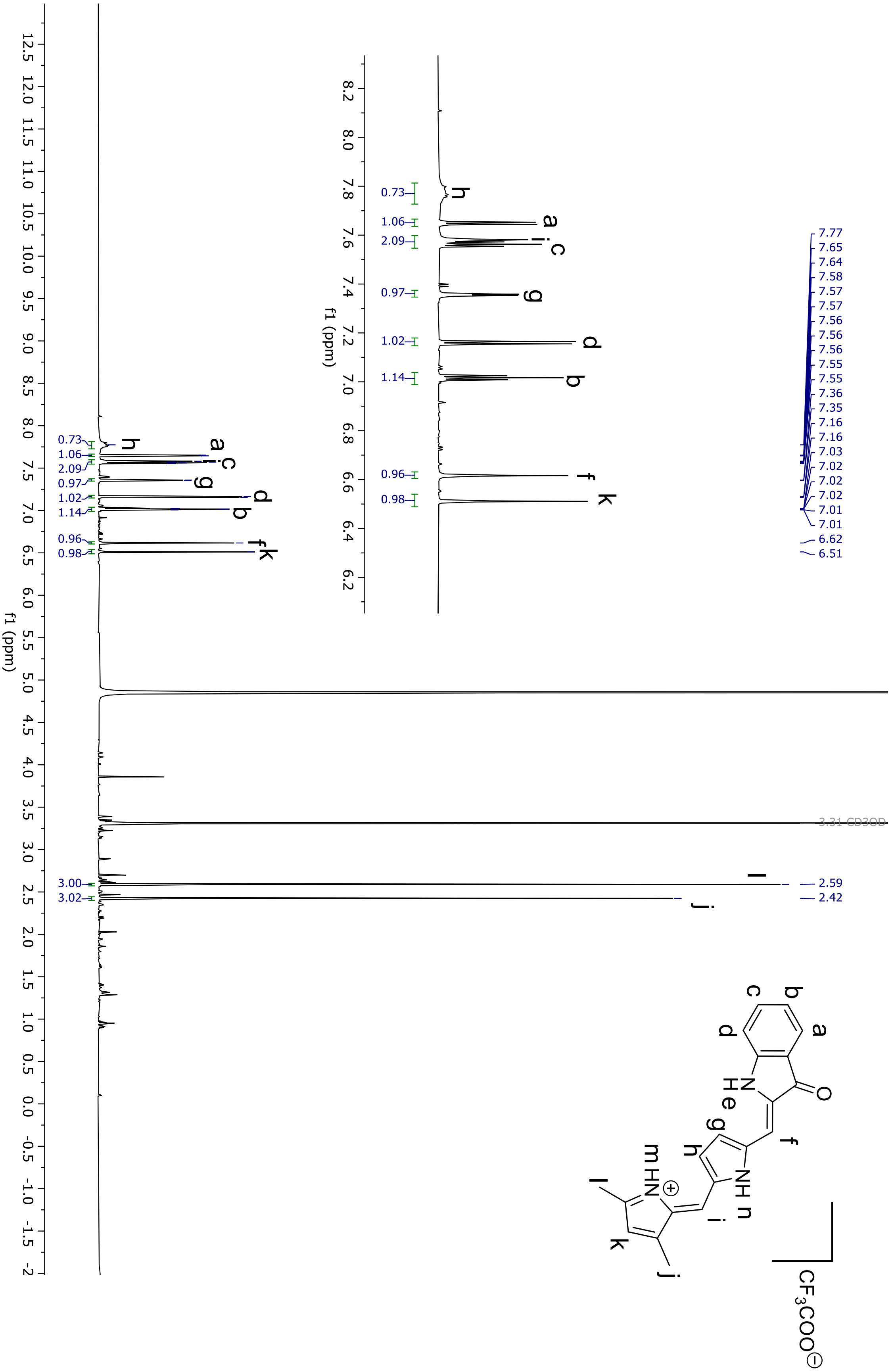

Figure S1. ^{1}H NMR spectrum (850 MHz, MeOD) of (*Z*)-**1**·TFA measured at 298 K.

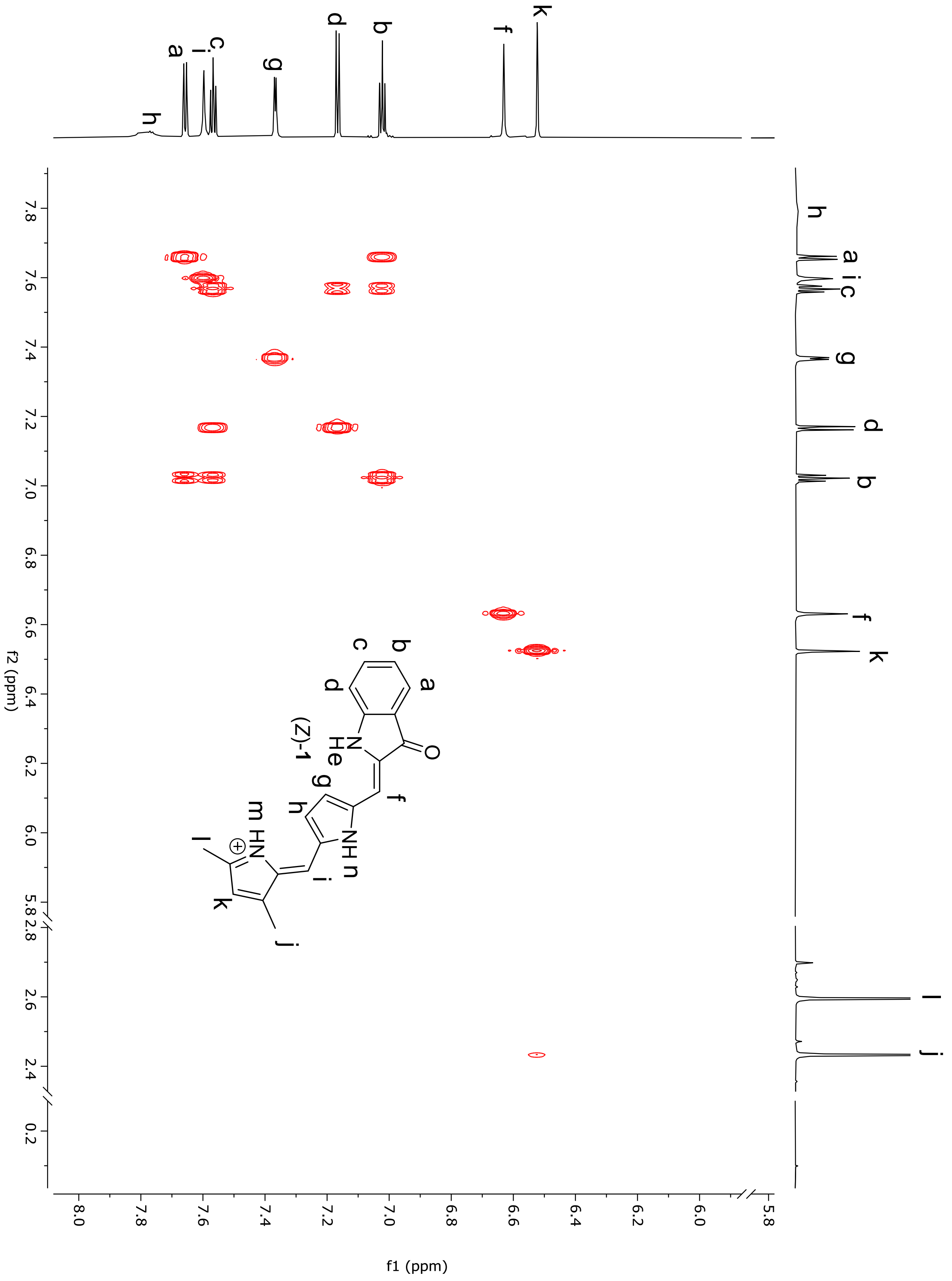

Figure S2. ^{1}H COSY spectrum (850 MHz, MeOD) of (Z)-**1**·TFA measured at 298 K

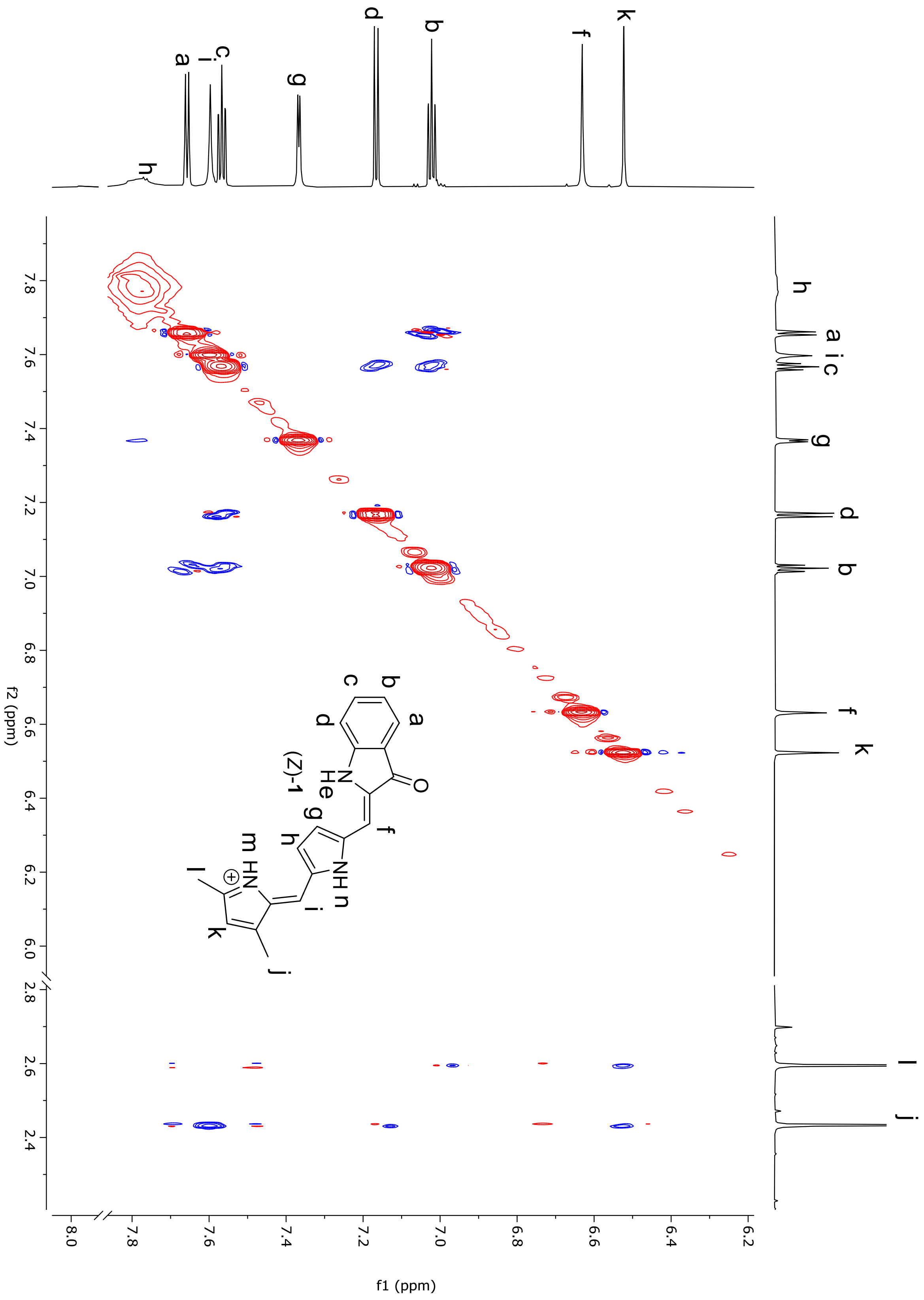

Figure S3. ^{1}H NOESY spectrum (850 MHz, MeOD) of (Z)-**1**·TFA measured at 298 K

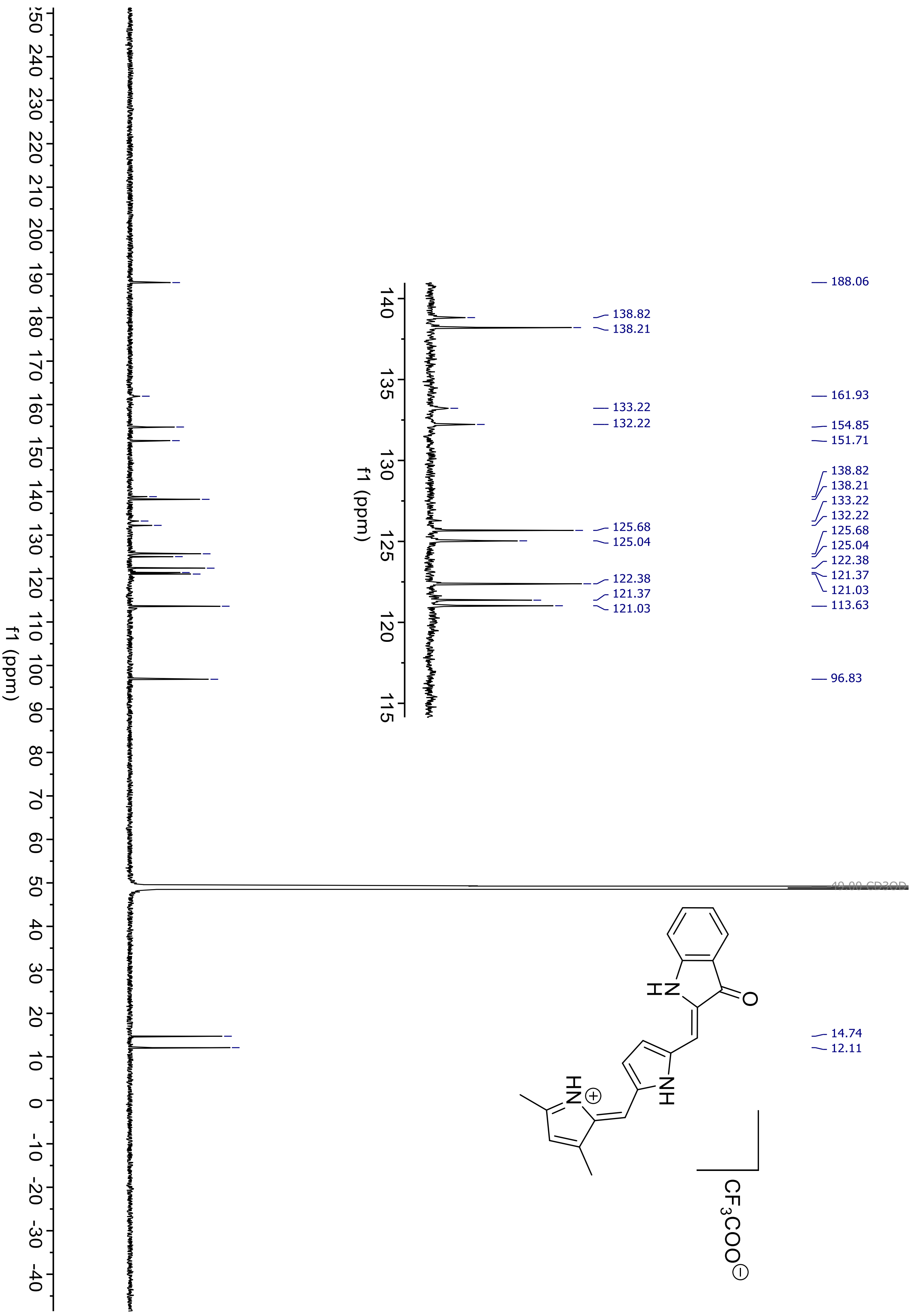

Figure S4. ^{13}C NMR spectrum (214 MHz, MeOD) of (*Z*)-**1**·TFA measured at 298 K.

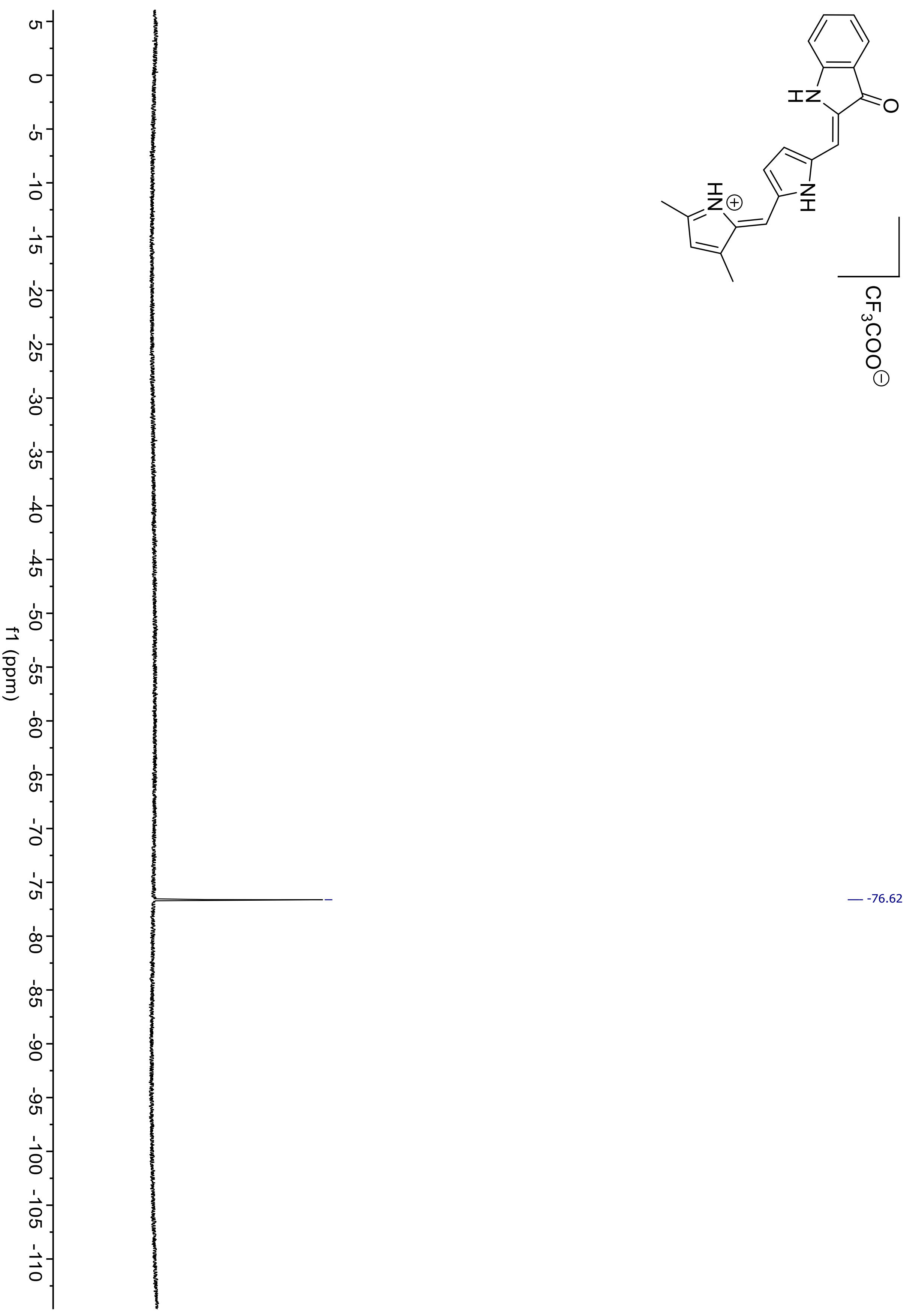

Figure S5. ^{19}F NMR spectrum (376 MHz, MeOD) of (*Z*)-**1**·TFA measured at 298 K.

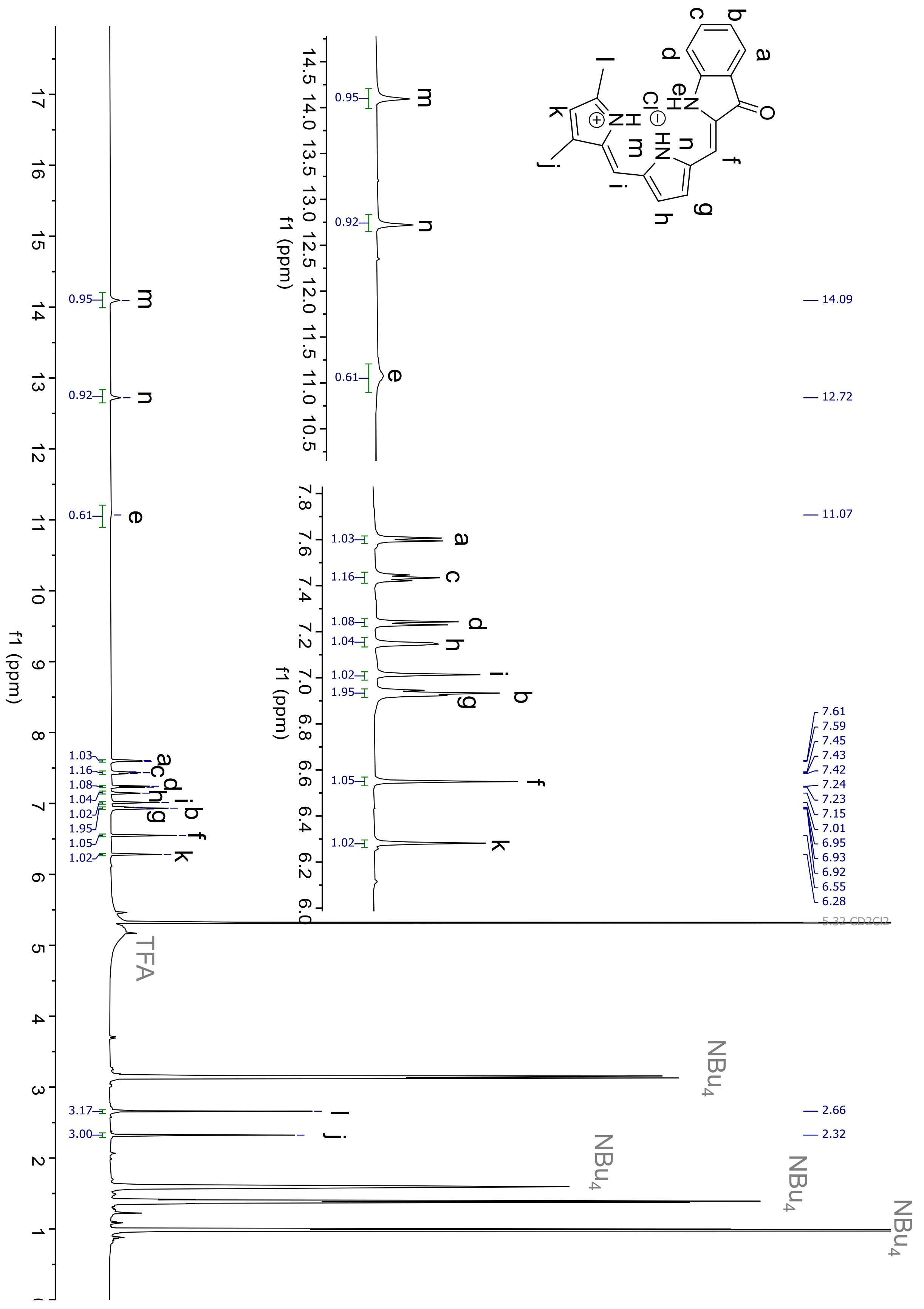

Figure S6. ^{1}H NMR spectrum (600 MHz, CD_2Cl_2) of (*Z*)-**1**·TFA measured at 263 K in presence of 2 equiv. NBu_4Cl and 2 equiv. TFA.

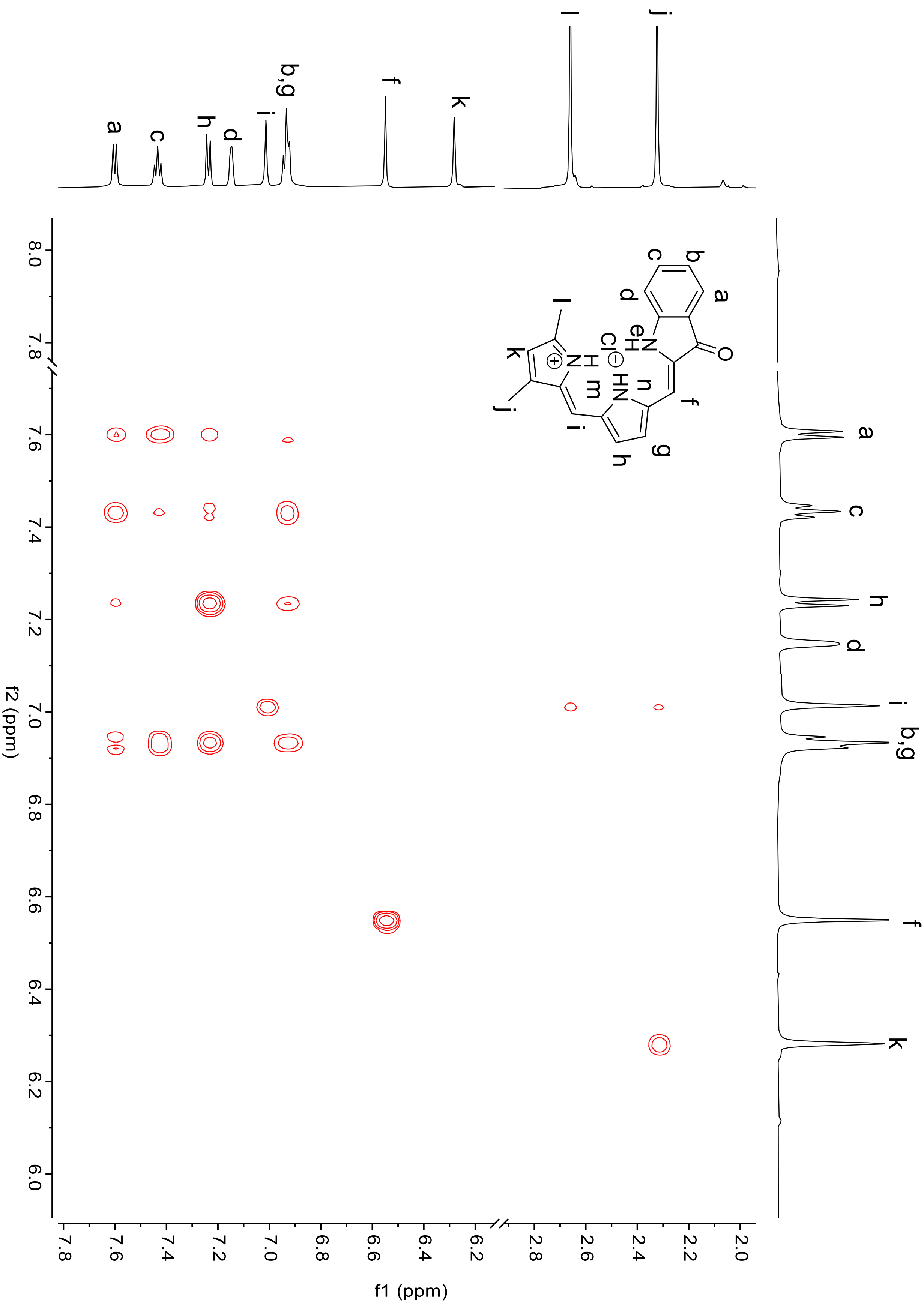

Figure S7. ^{1}H COSY spectrum (600 MHz, CD_2Cl_2) of (Z)-**1**·TFA measured at 263 K in presence of 2 equiv. NBu_4Cl and 2 equiv. TFA.

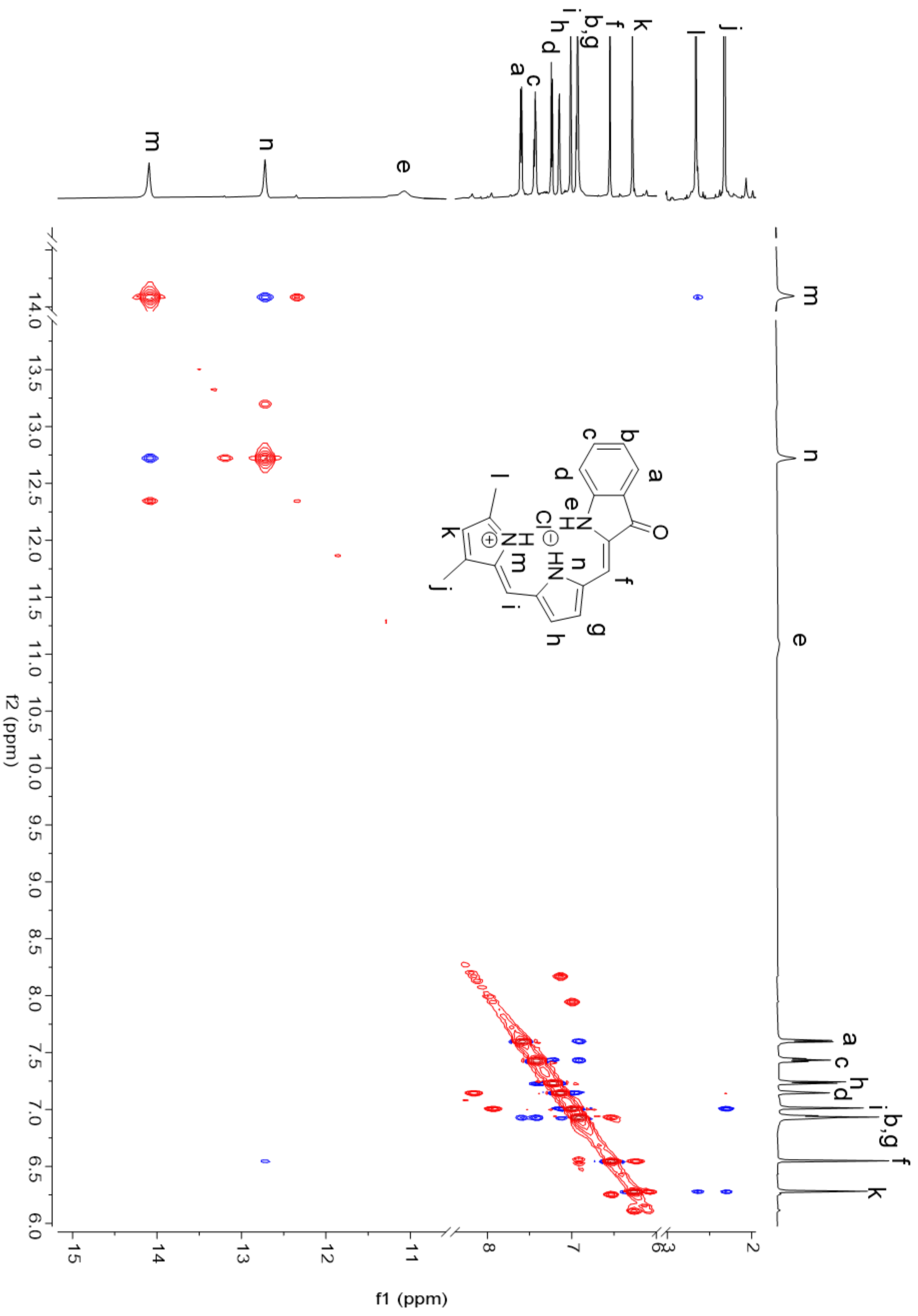

Figure S8. ^{1}H NOESY spectrum (600 MHz, CD_2Cl_2) of (Z)-**1**·TFA measured at 263 K in presence of 2 equiv. NBu_4Cl and 2 equiv. TFA.

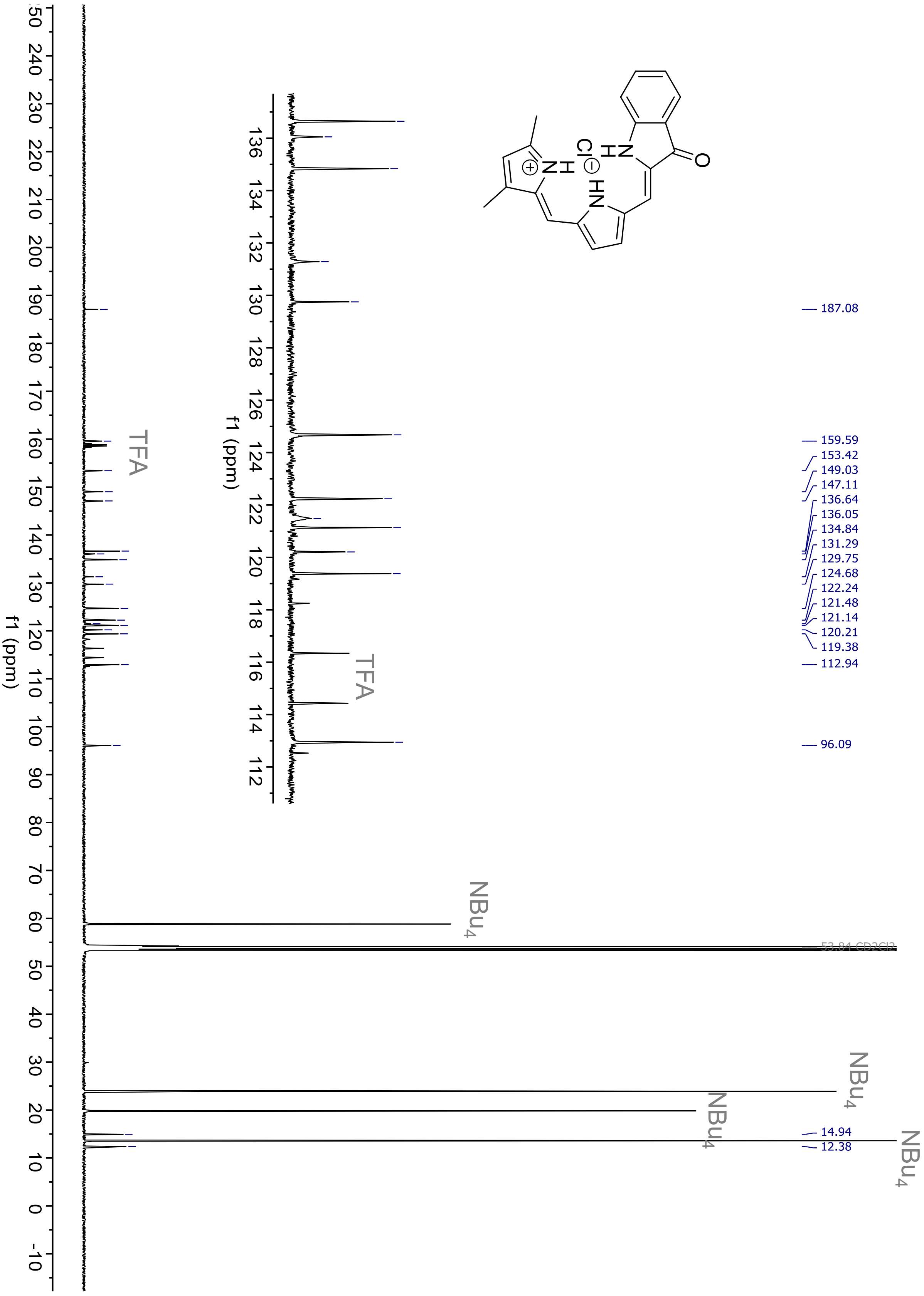

Figure S9. ^{13}C NMR (214 MHz, CD_2Cl_2) of (*Z*)-**1**·TFA measured at 263 K in presence of 2 equiv. NBu_4Cl and 2 equiv. TFA.

3. UV-Vis photoisomerization studies

Note: The amount of TFA and Et_3N added in these studies was chosen as such that no further spectral changes were observed after the addition of more equivalents.

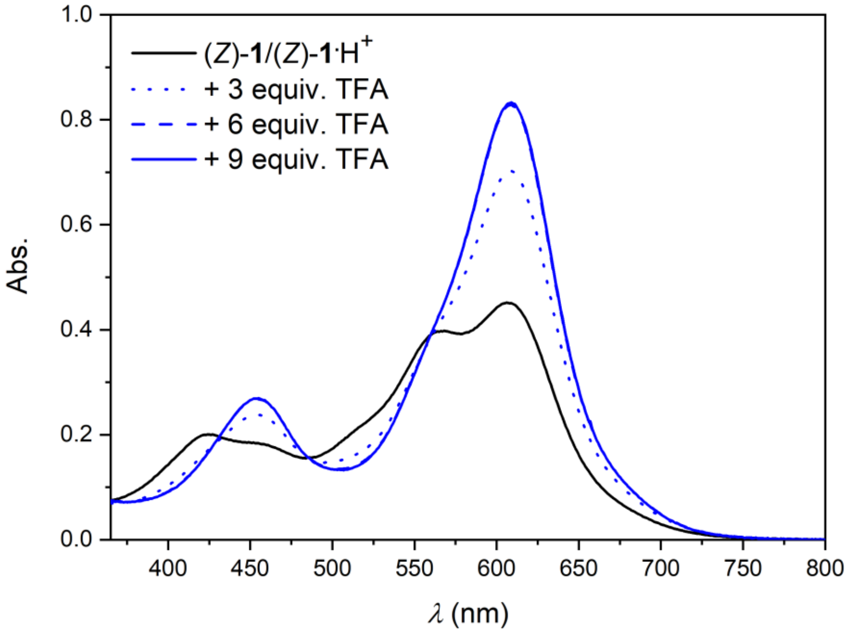

Figure S10. UV-Vis spectral changes of a solution of as-isolated (*Z*)-**1**·TFA (15 μM in MeOH) upon addition of more TFA (0.05 M in MeOH).

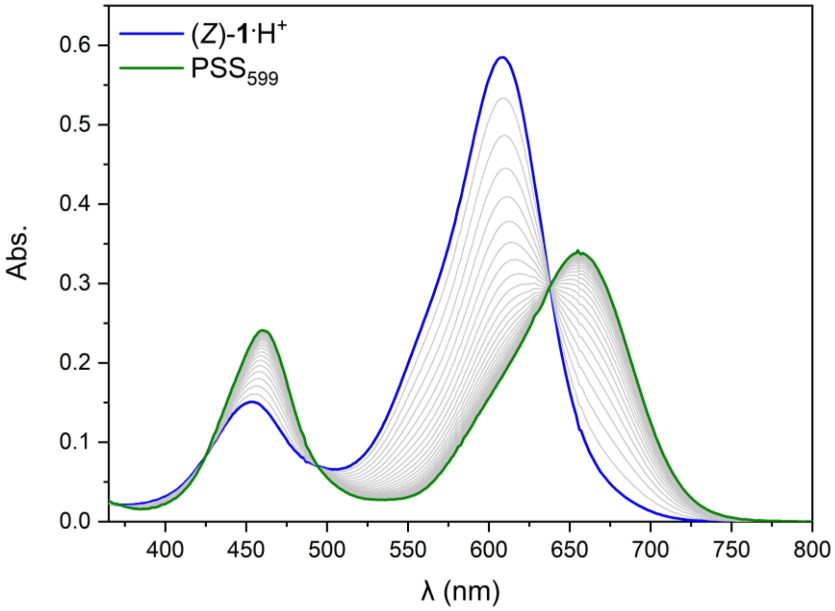

Figure S11. UV-Vis spectral changes during irradiation of (*Z*)-**1**·H$^+$ (10 μM in MeOH, 12 equiv. of TFA) with 599 nm light (60 s interval) showing a clear isosbestic point at λ = 638 nm.

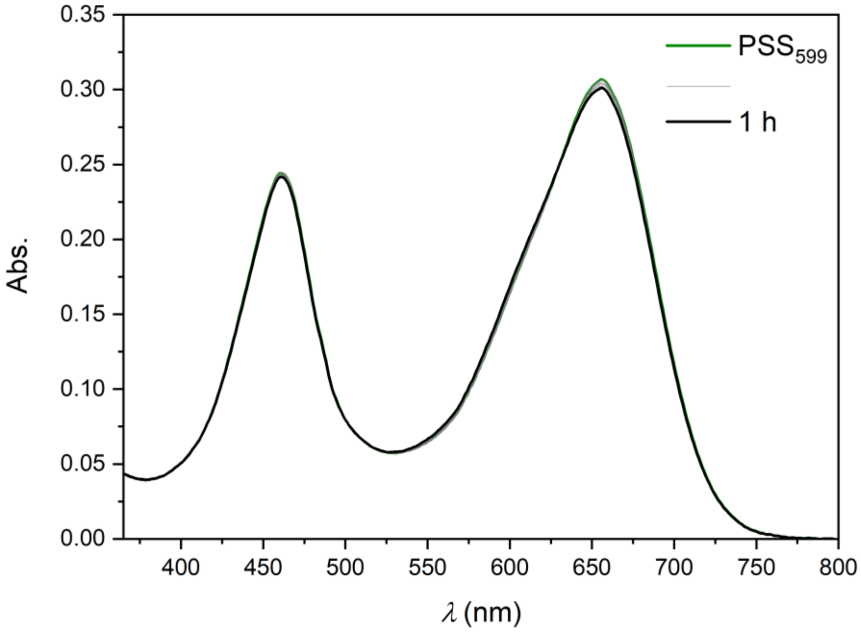

Figure S12. UV-Vis spectra of (E_{PSS})-**1**·H$^+$ (10 μM in MeOH, 12 equiv. of TFA) in the dark (600 s interval); ΔAbs. (at 655 nm) ≤ 2% after 1 h, highlighting the thermal stability.

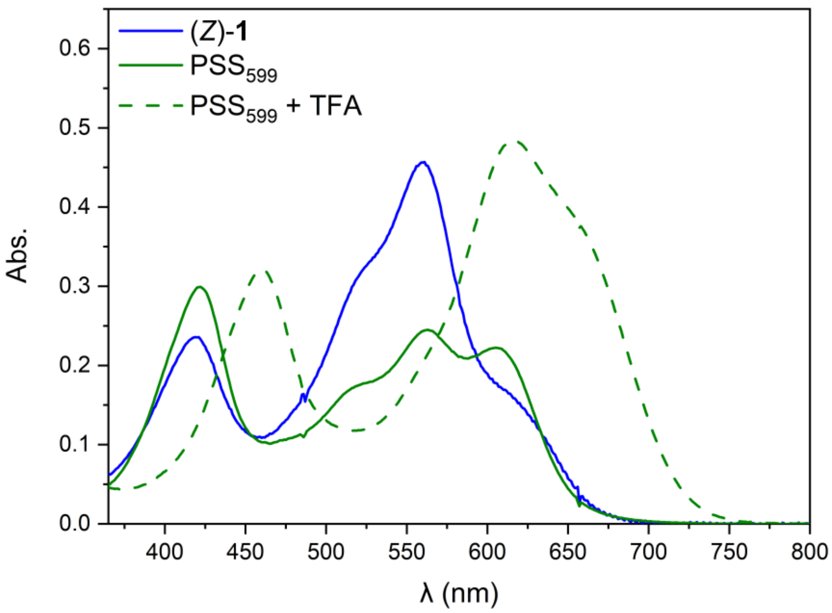

Figure S13. UV-Vis spectral changes of (*Z*)-**1** (15 μM in MeOH, 3 equiv. Et_3N, 273 K) upon irradiation with 599 nm light until the PSS was reached and after reacidification with TFA (20 equiv.).

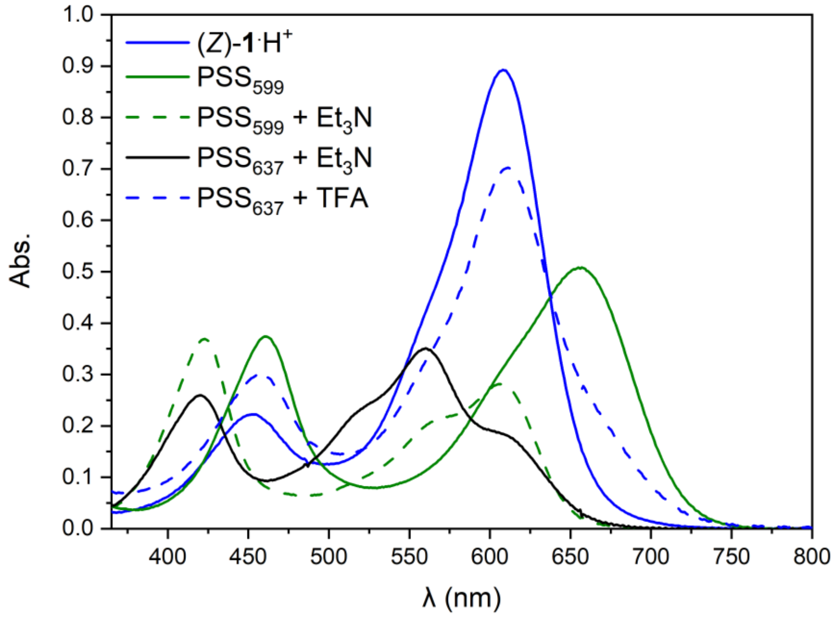

Figure S14. UV-Vis spectral changes of (*Z*)-**1**·H$^+$ (15 μM in MeOH, 3 equiv. TFA, 273 K) upon irradiation with 599 nm light until the PSS was reached and after basification with Et_3N (20 equiv.). Subsequently, the basified PSS_{599} was irradiated with 637 nm light until a new PSS was reached, after which the sample was acidified with TFA (40 equiv.).

Note: The neutral form of both isomers of **1** is expected to equilibrate between multiple tautomeric forms. Indeed, spectral changes were observed over time that are best accounted for by tautomerization. The given spectra thus belong to isomer mixtures, but identification of these mixtures is beyond the scope of this work. In the protonated form, which we study herein, these tautomeric forms are degenerate.

4. ^{1}H NMR photoisomerization studies

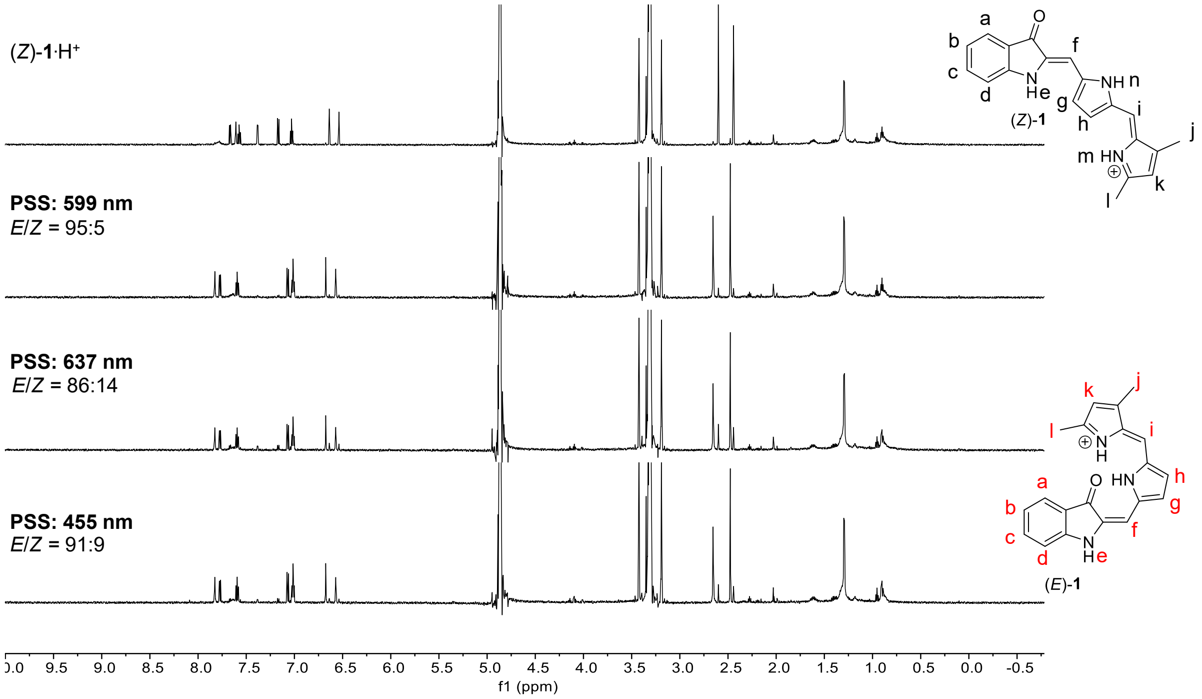

Figure S15. ^{1}H NMR spectra (600 MHz, MeOD) of (*Z*)-**1**·H^{+} (0.15 mM, 10 equiv. TFA) upon sequential irradiation with 599 nm, 637 nm, and 455 nm light. The integrals of signals H**l** and H**j** were used to calculate the PSS ratios. For signal assignments see the amplification of the aromatic and aliphatic region in Figures S16 and S17, respectively.

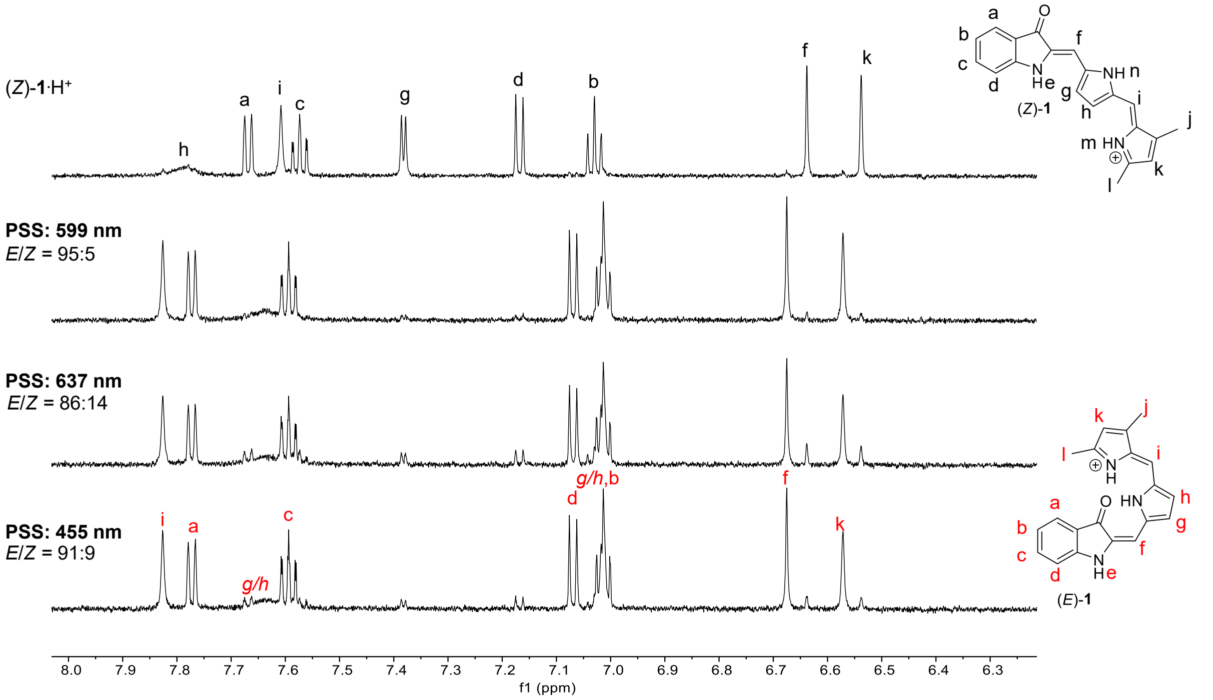

Figure S16. Aromatic region in the ^{1}H NMR spectra (600 MHz, MeOD) of (*Z*)-**1**·H^{+} (0.15 mM, 10 equiv. TFA) upon sequential irradiation with 599 nm, 637 nm and 455 nm light. The integrals of signals H**l** and H**j** were used to calculate the PSS ratios.

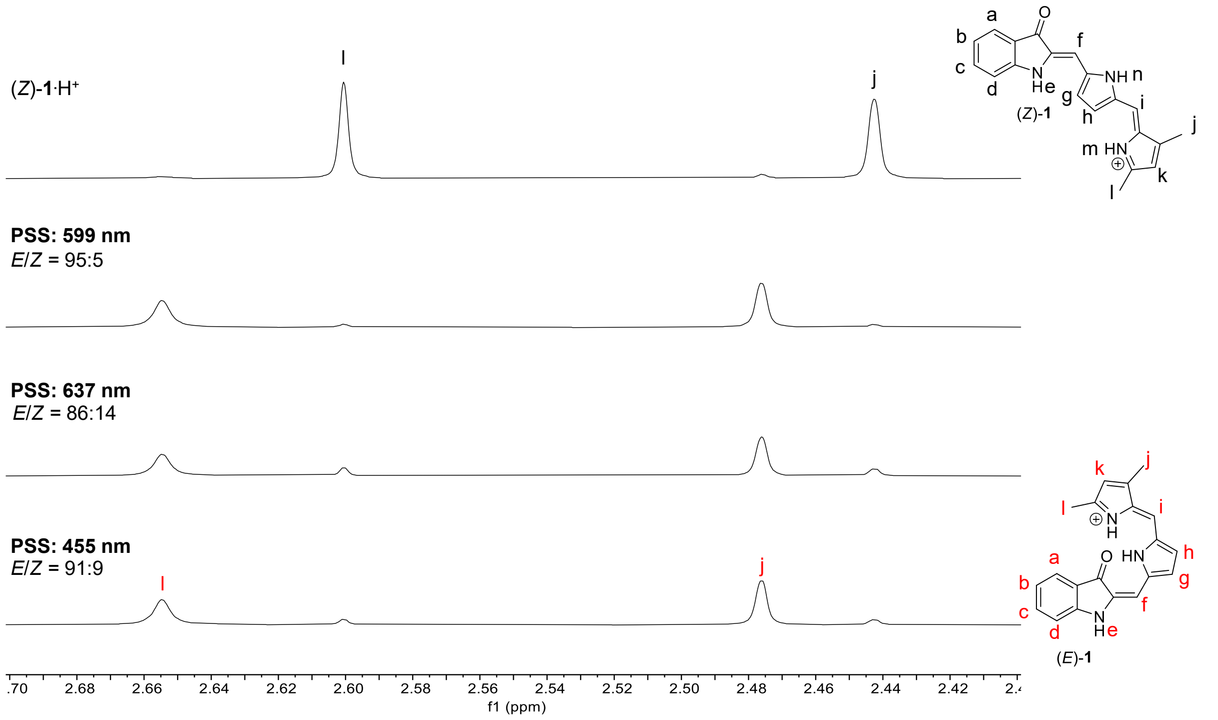

Figure S17. Aliphatic region in the ^{1}H NMR spectra (600 MHz, MeOD) of (*Z*)-**1**·H^{+} (0.15 mM, 10 equiv. TFA) upon sequential irradiation with 599 nm, 637 nm and 455 nm light. The integrals of signals H**l** and H**j** were used to calculate the PSS ratios.

5. ^{1}H NMR titration studies

Prior to titration experiments 10 mg aliquots of (Z)-**1**·TFA were additionally purified using FC (C_{18} SiO_2; CH_3CN:H_2O 2:8 to 8:2 with 0.1v% TFA) and freeze-dried. First, the as-isolated (*Z*)-**1**·TFA was dissolved in either MeOD or CD_2Cl_2/MeOD (95:5 v/v) to give a 1 mM solution. The (*E*)-isomer was generated by irradiation of the (*Z*)-isomer in MeOD (1 mM) with 599 nm light. This solution was used as is or concentrated and redissolved in CD_2Cl_2/MeOD (95:5 v/v) to a concentration of 1 mM. Separate solutions of NBu_4Cl (100 mM or 1.0 M) were prepared in the same solvent (mixture). The titrations were performed in presence of additional trifluoracetic acid [2 equiv. TFA in MeOD and 4 equiv. TFA in CD_2Cl_2/MeOD (95:5 v/v)] to ensure full protonation. Small aliquots of the guest solution were added to the receptor solution, and after each addition a ^{1}H NMR spectrum was recorded.

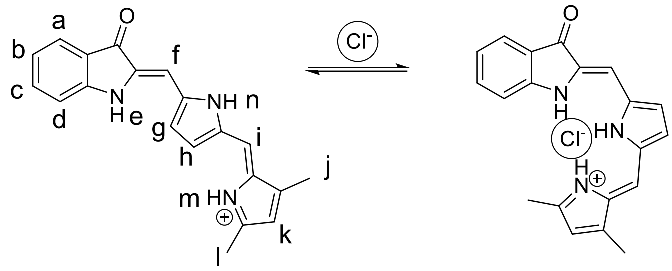

Scheme S2. Scheme of binding equilibrium including the lettering assignment of (*Z*)-**1**·H^{+}.

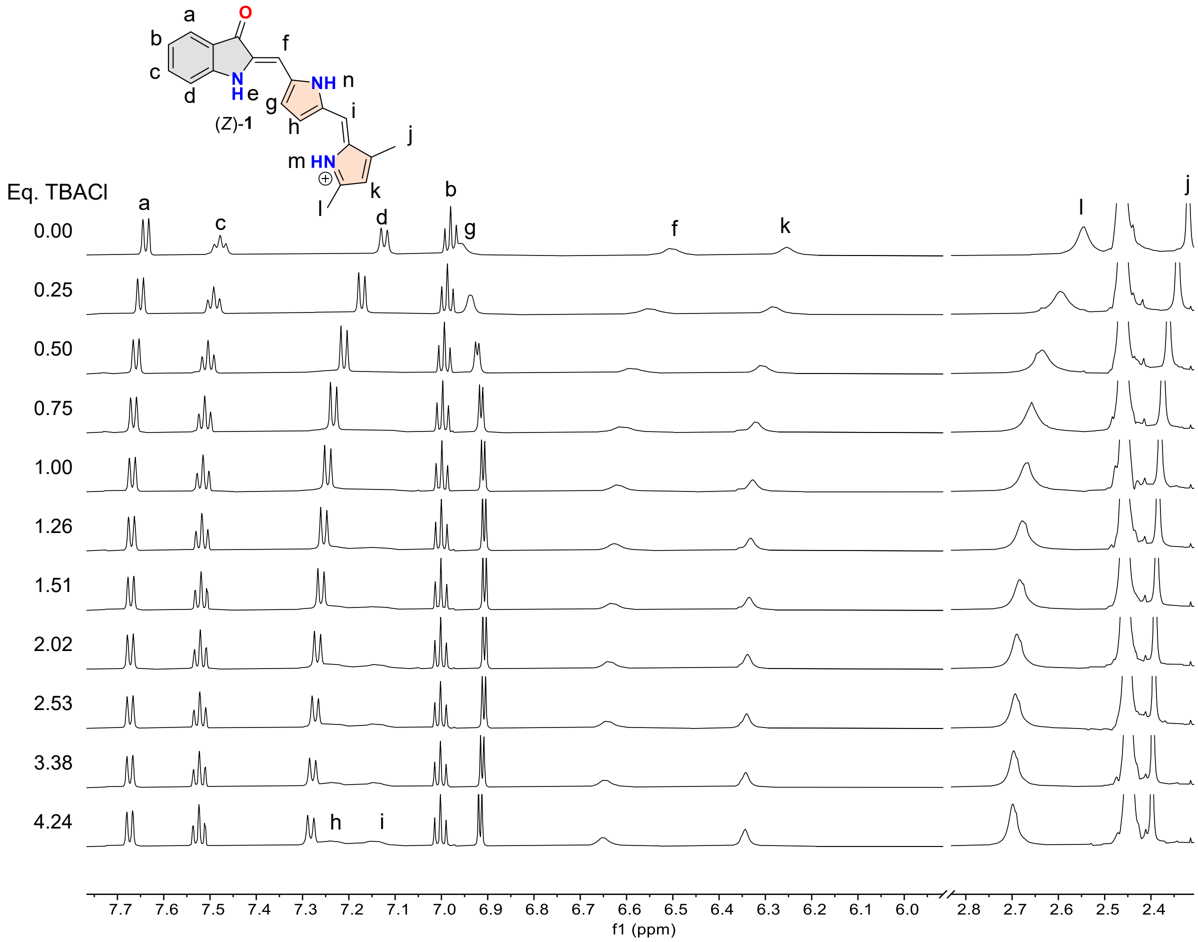

Figure S18. ^{1}H NMR spectra (600 MHz, CD_2Cl_2/MeOD 95:5 v/v) of (*Z*)-**1**·H^+ (1 mM, 4 equiv. of TFA) upon incremental addition of NBu_4Cl (100 mM).

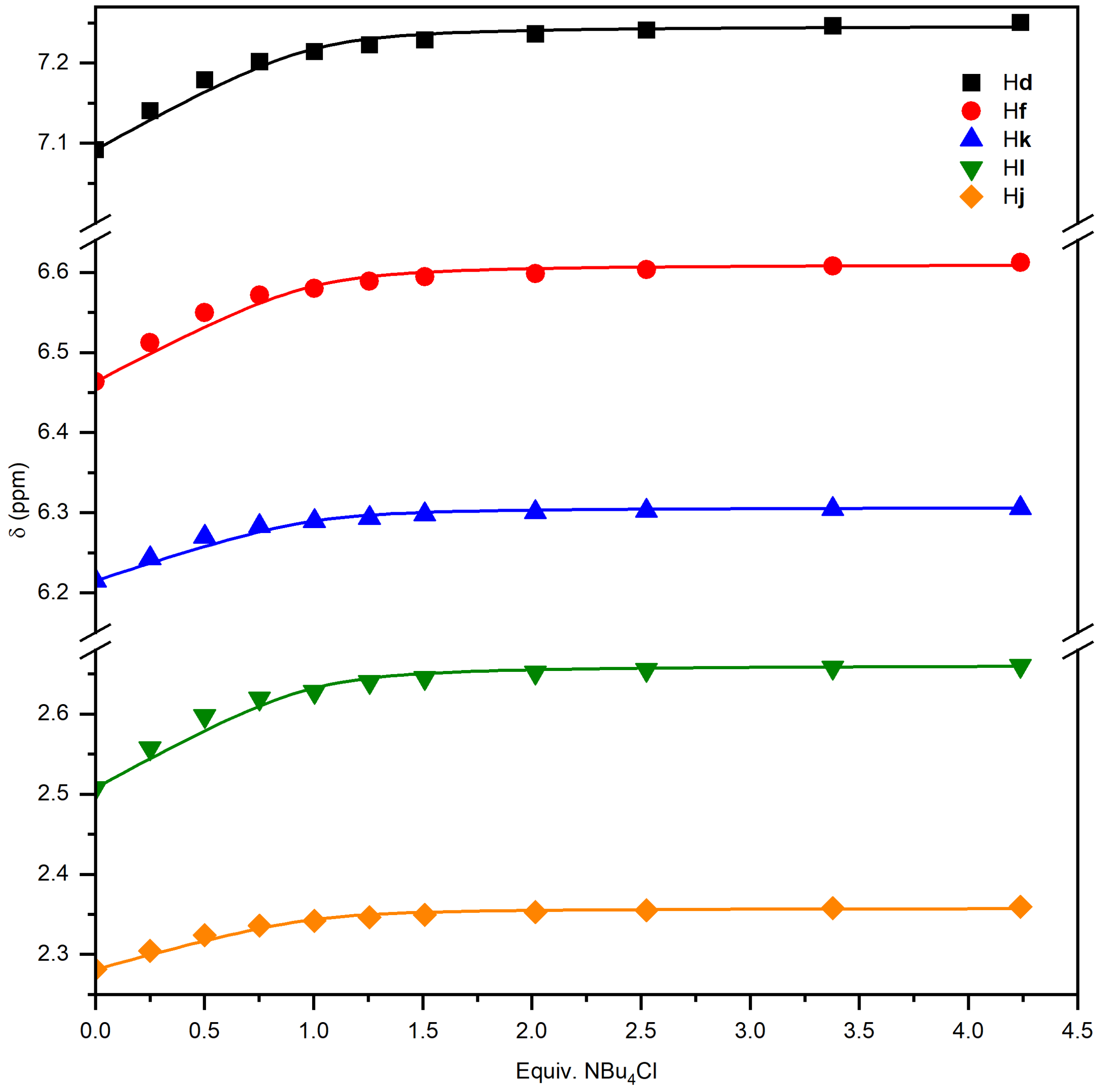

Figure S19. Titration curves for the addition of NBu_4Cl to (*Z*)-**1**·H^+ in CD_2Cl_2/MeOD (95:5 v/v, see Figure S12), and data fits obtained by simultaneous analysis of the indicated proton signals using HypNMR[2] and a 1:1 binding model; log(K_a) = 4.37 ± 0.127, or K_a = (2.4 ± 0.7)× 10^4 M^{-1}.

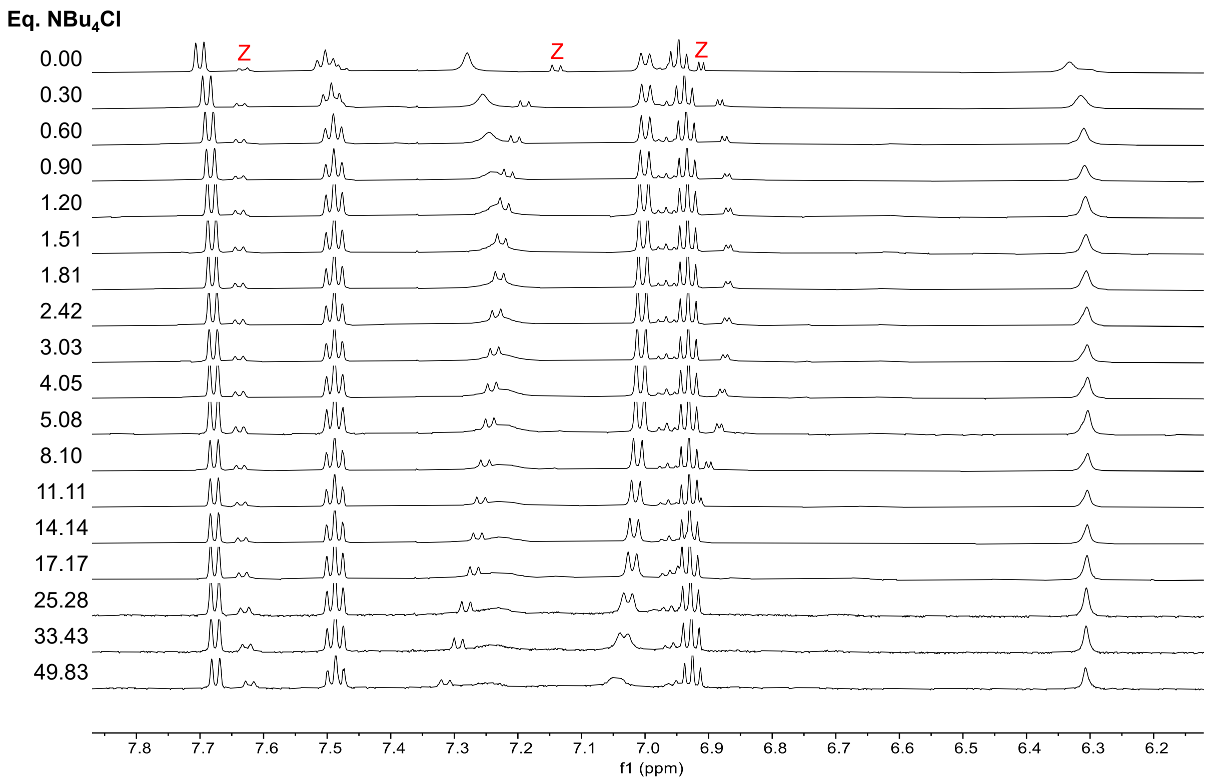

Figure S20. ^{1}H NMR spectra (600 MHz, CD_2Cl_2/MeOD 95:5 v/v) of (E_{PSS})-**1**·H^+ (1 mM, 4 equiv. of TFA) upon incremental addition of NBu_4Cl (100 mM). By relative ^{1}H NMR signal integration, an *E*/*Z* ratio of 16:84 was determined at the start of the titration.

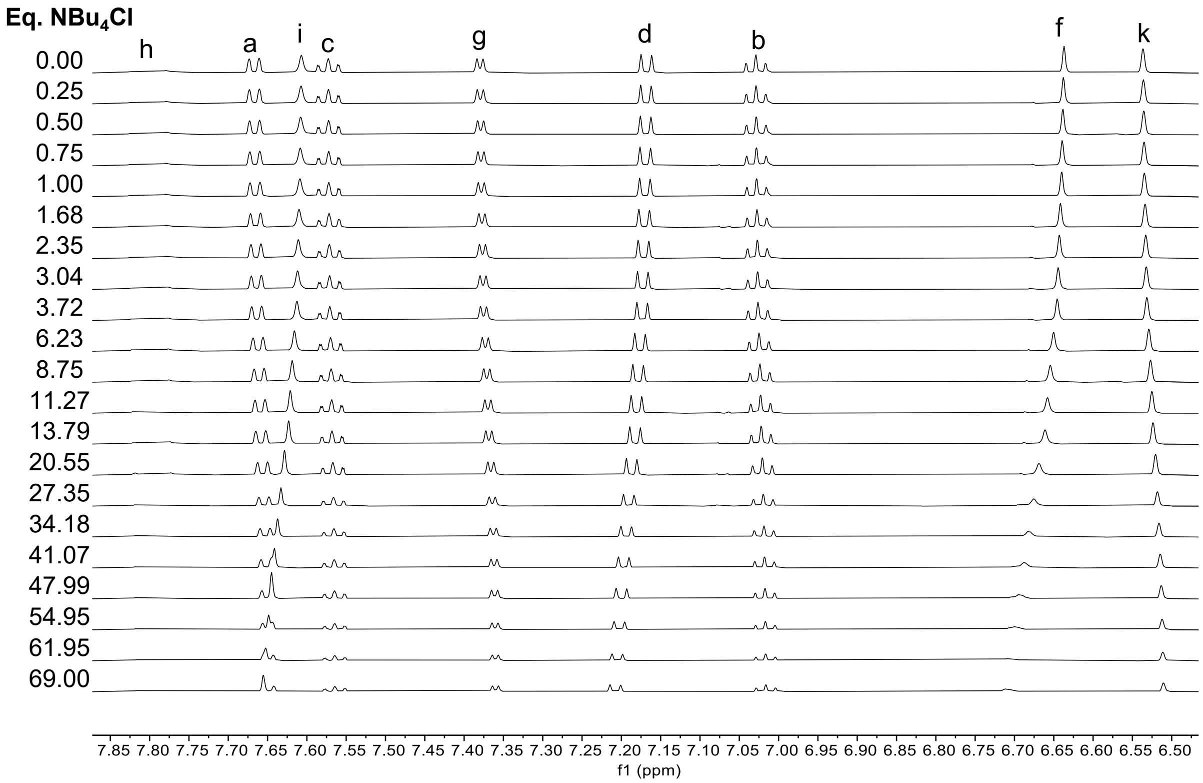

Figure S21. ^{1}H NMR spectra (600 MHz, MeOD) of (*Z*)-**1**·H^{+} (1 mM, 2 equiv. TFA) upon incremental addition of NBu_4Cl (100 mM and 1.0 M).

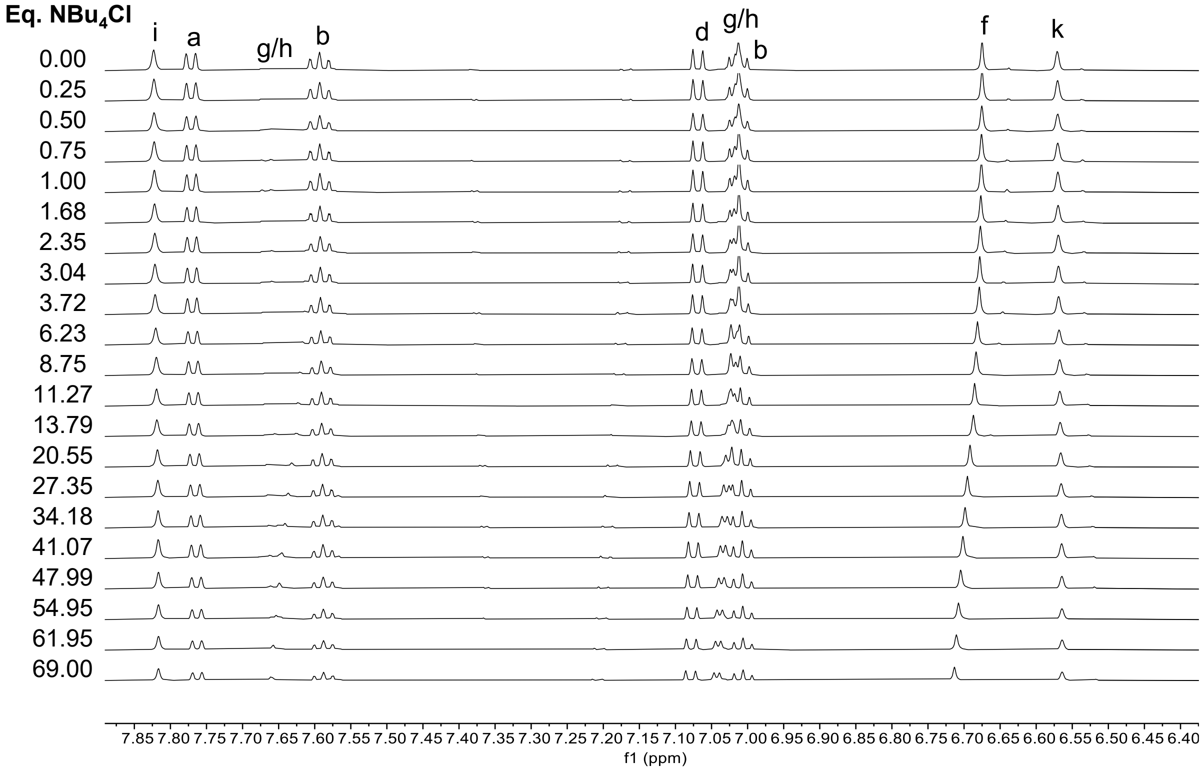

Figure S22. ^{1}H NMR spectra (600 MHz, MeOD) of (E_{PSS})-**1**·H^{+} (1 mM, 2 equiv. TFA) upon incremental addition of NBu_4Cl (100 mM and 1.0 M).

6. Cationophore-coupled ISE assays

The ISE transport assay was performed according to the procedure previously described by the group of Gale.[3] A lipid film was prepared by evaporating a solution of POPC in $CHCl_3$ in a round-bottom flask, followed by drying under high vacuum for at least 12 h. The lipid film was hydrated with an aqueous solution of 300 mM KCl buffered with either HEPES (pH 7.2, 5 mM) or MES (pH 5.5, 5 mM) and the resulting suspension was vortexed. Subsequently, the suspension was subjected to 9 freeze-thaw cycles, by freezing the sample in liquid nitrogen and thawing in a water bath (45 °C). The sample was allowed to equilibrate for 30 minutes, after which it was extruded 25 times through a 200 nm polycarbonate membrane to obtain unilamellar vesicles. An Illustra NAP™-25 Sephadex® G-25 column was equilibrated with an aqueous solution of 300 mM KGlu buffered with either HEPES (pH 7.2, 5 mM) or MES (pH 5.5, 5 mM) and the liposome suspension was eluted on this column using the aqueous KGlu solution to exchange KCl for KGlu.

Prior to the measurement the ISE chloride selective electrode was calibrated using a series of solutions with a known chloride concentration: 0.159 mM, 0.318 mM, 0.625 mM, 1.25 mM, 2.50 mM, 5.00 mM, and 10.0 mM. By fitting the calibration data to formula S**1** constants *a* and *b* could be obtained.

$$E\ (\mathrm{mV}) = \mathrm{a} + \mathrm{b}\log[\mathrm{Cl}] \qquad (\mathrm{S}\mathbf{1})$$

Subsequently, a measured value of *E* (mV) could be converted into [Cl] by using formula S**2**.

$$[\mathrm{Cl}] = 10^{\left(\frac{E-\mathrm{a}}{\mathrm{b}}\right)} \qquad (\mathrm{S}\mathbf{2})$$

For each measurement the liposome solution was diluted to 1.0 mM in the aqueous solution of 300 mM KGlu buffered with either HEPES (pH 7.2, 5 mM) or MES (pH 5.5, 5 mM) in a glass vial. Chloride efflux was measured over a time course of 8 minutes, with a data interval of 3 s.

At t = 15 s either MeOH (blank) or a solution of the transporter in MeOH was added, and after t = 30 s DMSO (blank) or a DMSO solution of either monensin or valinomycin (0.1 mol%, 5 µL) was added to initiate transport. After 6 minutes (t = 360 s) the liposomes were lysed with Triton-X (50 µL, 11 wt% in H_2O/DMSO 7:1 v/v) and after t = 480 s the final reading was taken.

The obtained changes in chloride concentration were normalized to give the percentage of chloride efflux using formula S**3**. All experiments were performed in duplicate.

$$\% \text{ Cl} = \frac{[\text{Cl}]_t - [\text{Cl}]_0}{[\text{Cl}]_{360} - [\text{Cl}]_0} \times 100 \qquad (\text{S}\mathbf{3})$$

Note: Prior to transport studies, 10 mg aliquots were additionally purified using FC (C_{18} SiO_2; CH_3CN:H_2O 2:8 to 8:2 with 0.1v% TFA). The assay was performed in the dark, to avoid photoisomerization during the execution of the assay. For the transport assays with the (*E*)-isomer, (E_{PSS})-**1**·H^+ was generated photochemically by irradiating a solution of (*Z*)-**1**·TFA in MeOH (1 mM) with 599 nm light (from here on referred to as (E_{PSS})-**1**). Photoisomerization was followed by UV-Vis spectroscopy to ensure the PSS was reached.

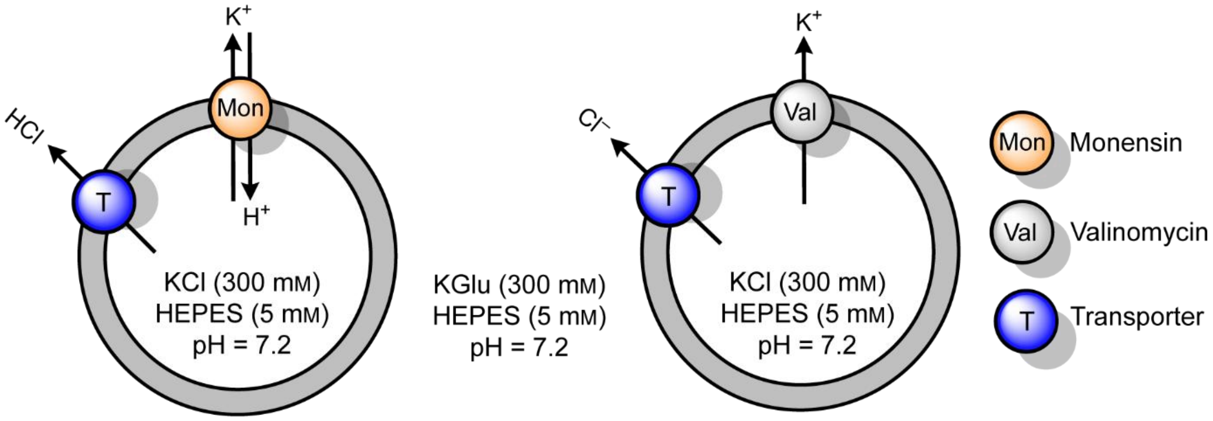

Figure S23. Schematic representation of monensin (Mon) and valinomycin (Val) coupled ISE assay to determine selectivity for either electroneutral (HCl) or electrogenic (Cl^-) transport mechanisms.

Selectivity assay of (*Z*)-1 at pH 7.2

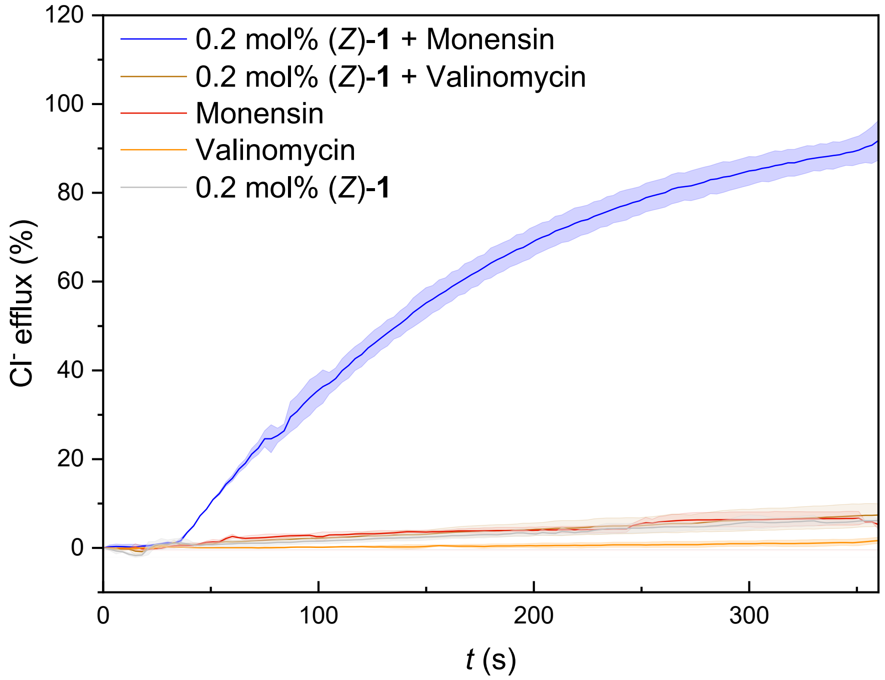

Figure S24. Chloride efflux plotted versus time as measured by a chloride selective electrode in the cationophore-coupled assay. The transporter was added from MeOH (1.0 mM stock solution) to the aqueous liposome solution (1.0 mM POPC, 300 mM internal KCl, 300 mM external KGlu buffered with HEPES to pH 7.2) at t = 15 s, followed by monensin (0.1 mol%) or valinomycin (0.1 mol%) from DMSO at t = 30 s, and an aqueous Triton-X solution at t = 360 s. Blank experiments were also performed in which either MeOH (without transporter) or DMSO (without cationophore) was added.

EC_{50} Determination of (*Z*)-1 at pH 7.2

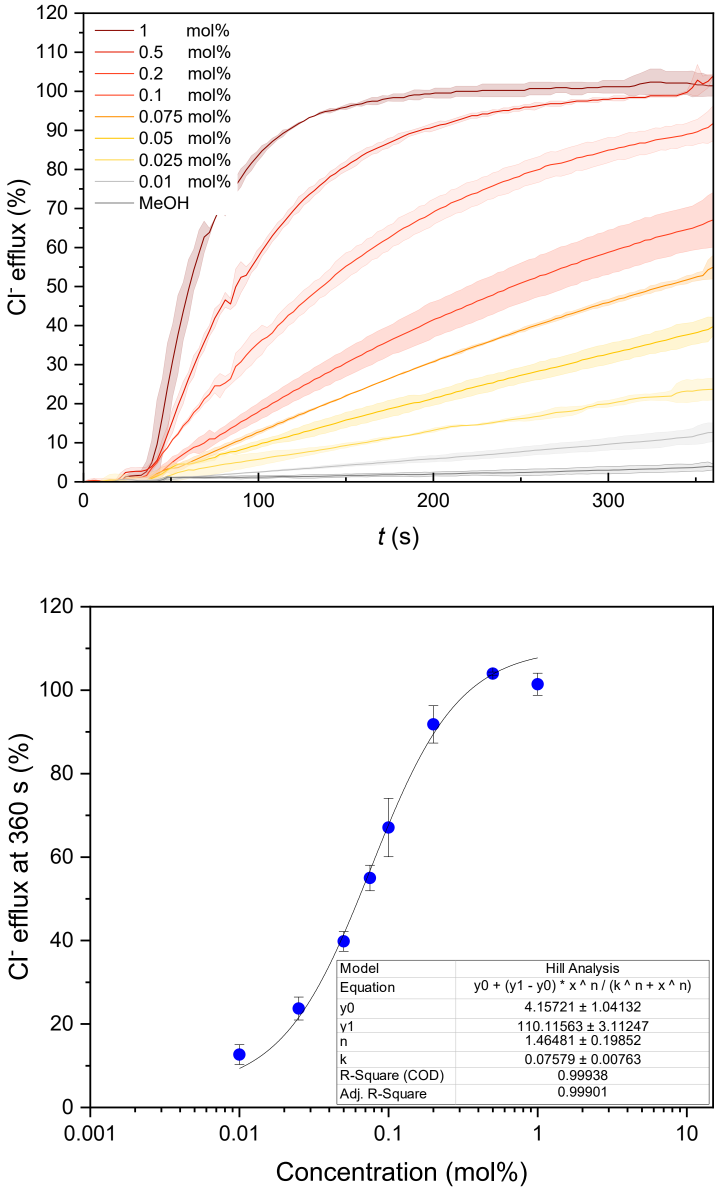

Figure S25. (Top) Chloride efflux plotted versus time as measured by a chloride selective electrode in the monensin-coupled assay. The transporter was added from MeOH (1.0 mM stock solution) to the aqueous liposome solution (1.0 mM POPC, 300 mM internal KCl, 300 mM external KGlu buffered with HEPES to pH 7.2) at $t = 15$ s, followed by monensin (0.1 mol%) from DMSO at $t = 30$ s, and an aqueous Triton-X solution at $t = 360$ s. Blank experiments were also performed in which MeOH (without transporter) was added. (Bottom) Chloride efflux at $t = 360$ s in the monensin-coupled ISE assay plotted versus concentration and fit to the Hill equation. The same data is shown in Figure 3B of the main text without details of the fit; $EC_{50} = 7.6 \times 10^{-2}$ mol% (with respect to lipids).

Selectivity assay of (*Z*)-1 at pH 5.5

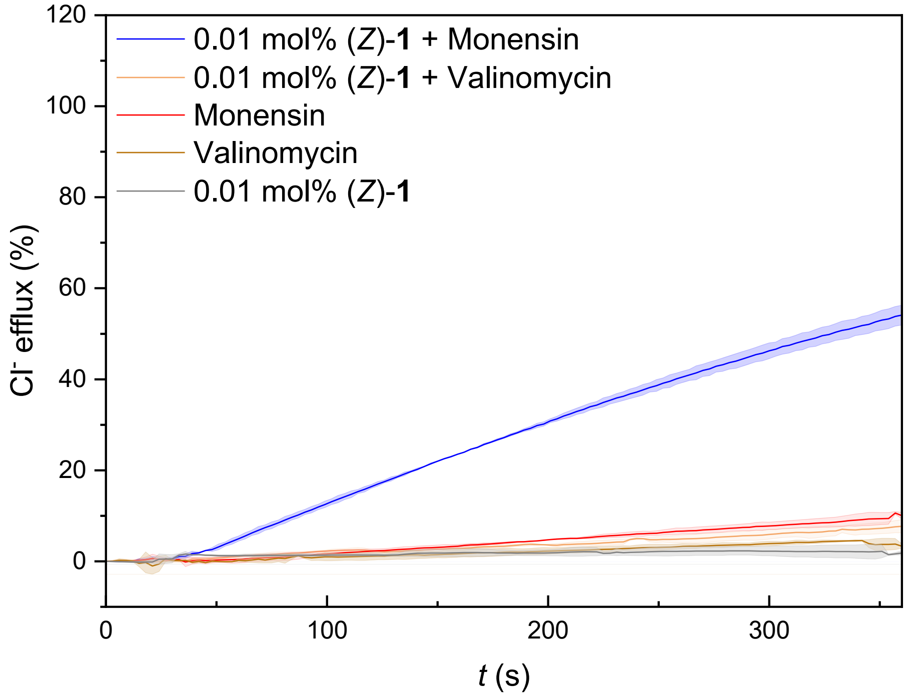

Figure S26. Chloride efflux plotted versus time as measured by a chloride selective electrode in the cationophore-coupled assay. The transporter was added from MeOH (1.0 mM stock solution) to the aqueous liposome solution (1.0 mM POPC, 300 mM internal KCl, 300 mM external KGlu buffered with MES to pH 5.5) at *t* = 15 s, followed by monensin (0.1 mol%) or valinomycin (0.1 mol%) from DMSO at *t* = 30 s, and an aqueous Triton-X solution at *t* = 360 s. Blank experiments were also performed in which either MeOH (without transporter) or DMSO (without cationophore) was added.

EC_{50} Determination of (*Z*)-1 at pH 5.5

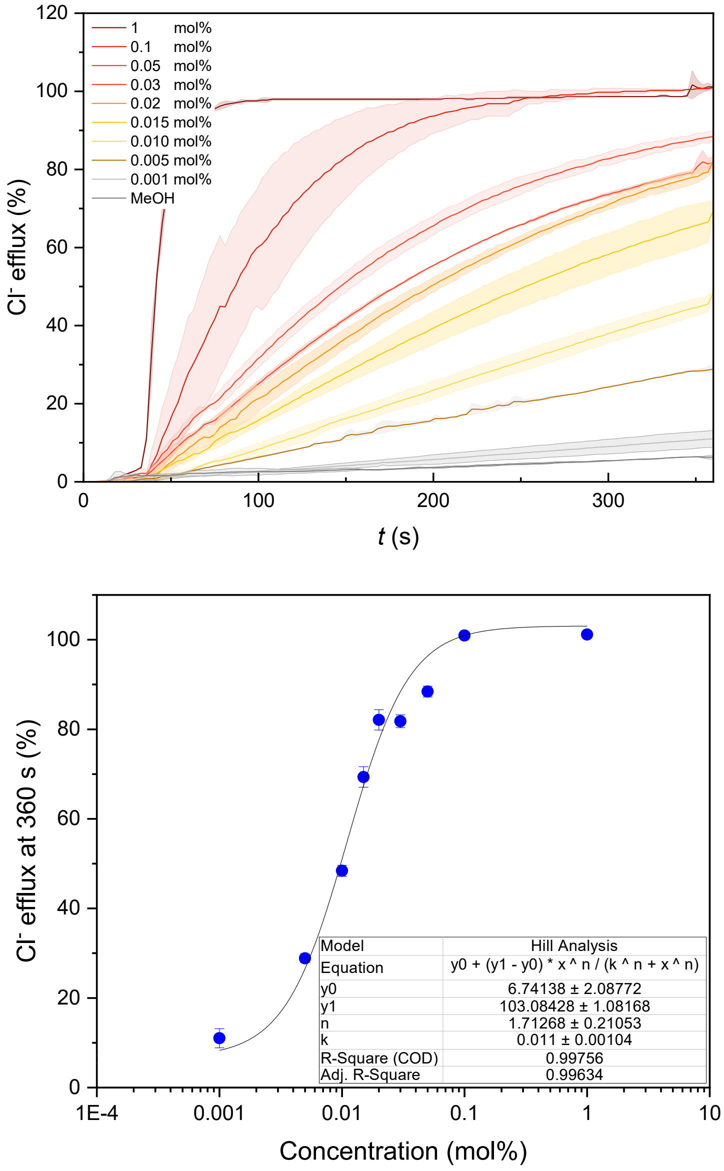

Figure S27. (Top) Chloride efflux plotted versus time as measured by a chloride selective electrode in the monensin-coupled assay. The transporter was added from MeOH (1.0 mM stock solution) to the aqueous liposome solution (1.0 mM POPC, 300 mM internal KCl, 300 mM external KGlu buffered with MES to pH 5.5) at t = 15 s, followed by monensin (0.1 mol%) from DMSO at t = 30 s, and an aqueous Triton-X solution at t = 360 s. Blank experiments were also performed in which MeOH (without transporter) was added. (Bottom) Chloride efflux at t = 360 s in the monensin-coupled ISE assay plotted versus concentration and fit to the Hill equation. The same data is shown in Figure 3B of the main text without details of the fit; EC_{50} = 1.1×10^{-2} mol% (with respect to lipids).

Selectivity assay of (E_{PSS})-1 at pH 7.2

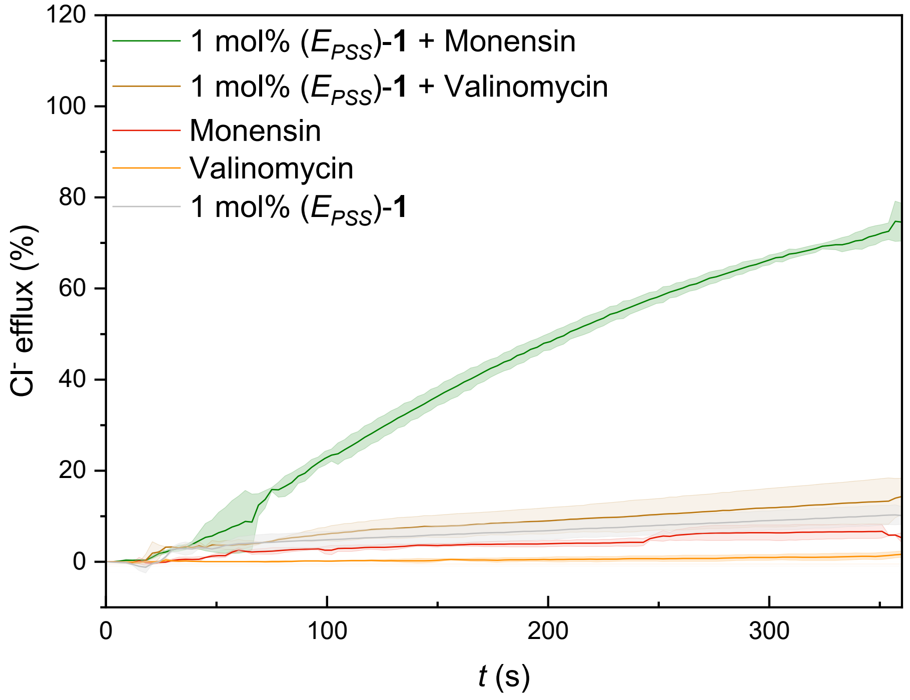

Figure S28. Chloride efflux plotted versus time as measured by a chloride selective electrode in the cationophore-coupled assay. The transporter was added from MeOH (1.0 mM stock solution) to the aqueous liposome solution (1.0 mM POPC, 300 mM internal KCl, 300 mM external KGlu buffered with HEPES to pH 7.2) at t = 15 s, followed by monensin (0.1 mol%) or valinomycin (0.1 mol%) from DMSO at t = 30 s, and an aqueous Triton-X solution at t = 360 s. Blank experiments were also performed in which either MeOH (without transporter) or DMSO (without cationophore) was added.

EC_{50} Determination of (E_{PSS})-1 at pH 7.2

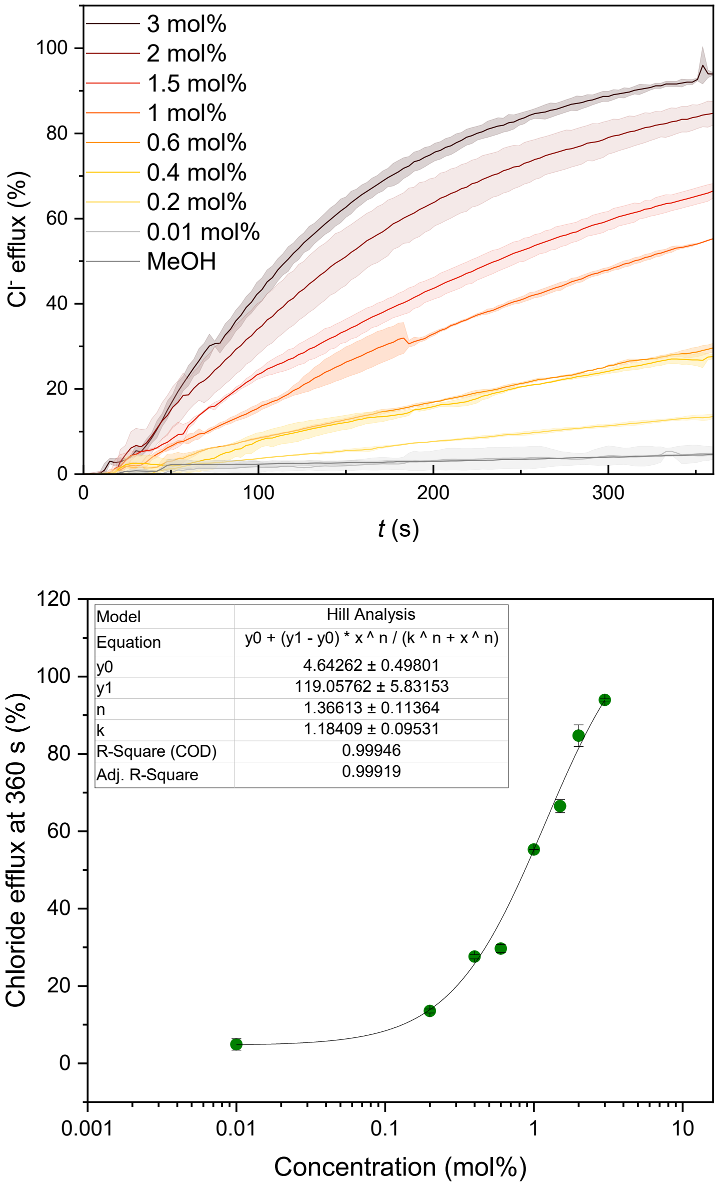

Figure S29. (Top) Chloride efflux plotted versus time as measured by a chloride selective electrode in the monensin-coupled assay. The transporter was added from MeOH (1.0 mM stock solution) to the aqueous liposome solution (1.0 mM POPC, 300 mM internal KCl, 300 mM external KGlu buffered with HEPES to pH 7.2) at t = 15 s, followed by monensin (0.1 mol%) from DMSO at t = 30 s, and an aqueous Triton-X solution at t = 360 s. Blank experiments were also performed in which MeOH (without transporter) was added. (Bottom) Chloride efflux at t = 360 s in the monensin-coupled ISE assay plotted versus concentration and fit to the Hill equation. The same data is shown in Figure 3B of the main text without details of the fit; EC_{50} = 1.18 mol% (with respect to lipids).

Selectivity assay of (E_{PSS})-1 at pH = 5.5

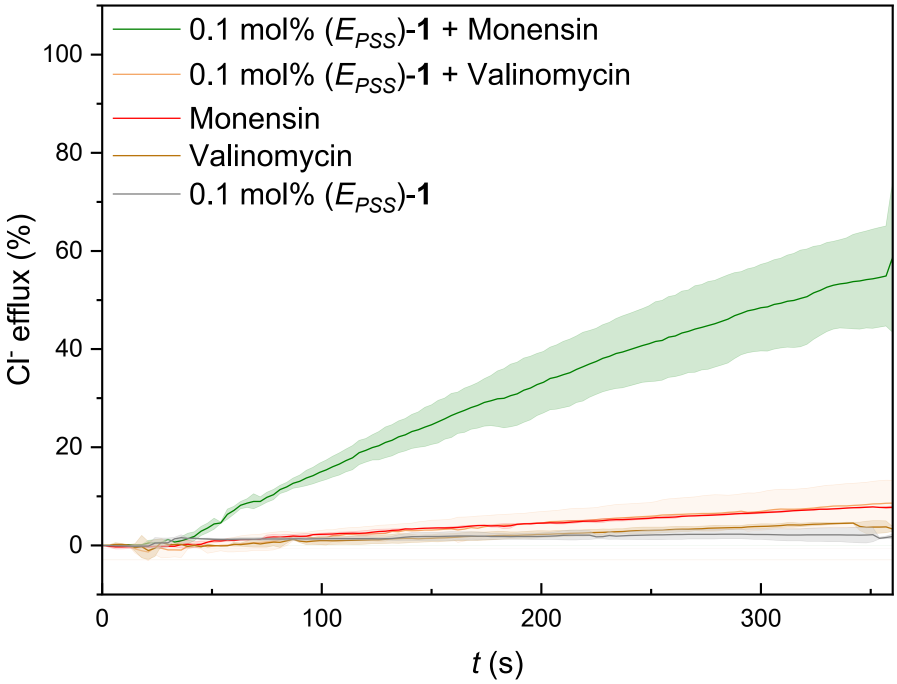

Figure S30. Chloride efflux plotted versus time as measured by a chloride selective electrode in the cationophore-coupled assay. The transporter was added from MeOH (1.0 mM stock solution) to the aqueous liposome solution (1.0 mM POPC, 300 mM internal KCl, 300 mM external KGlu buffered with MES to pH 5.5) at *t* = 15 s, followed by monensin (0.1 mol%) or valinomycin (0.1 mol%) from DMSO at *t* = 30 s, and an aqueous Triton-X solution at *t* = 360 s. Blank experiments were also performed in which either MeOH (without transporter) or DMSO (without cationophore) was added.

EC_{50} Determination of (E_{PSS})-1 at pH 5.5

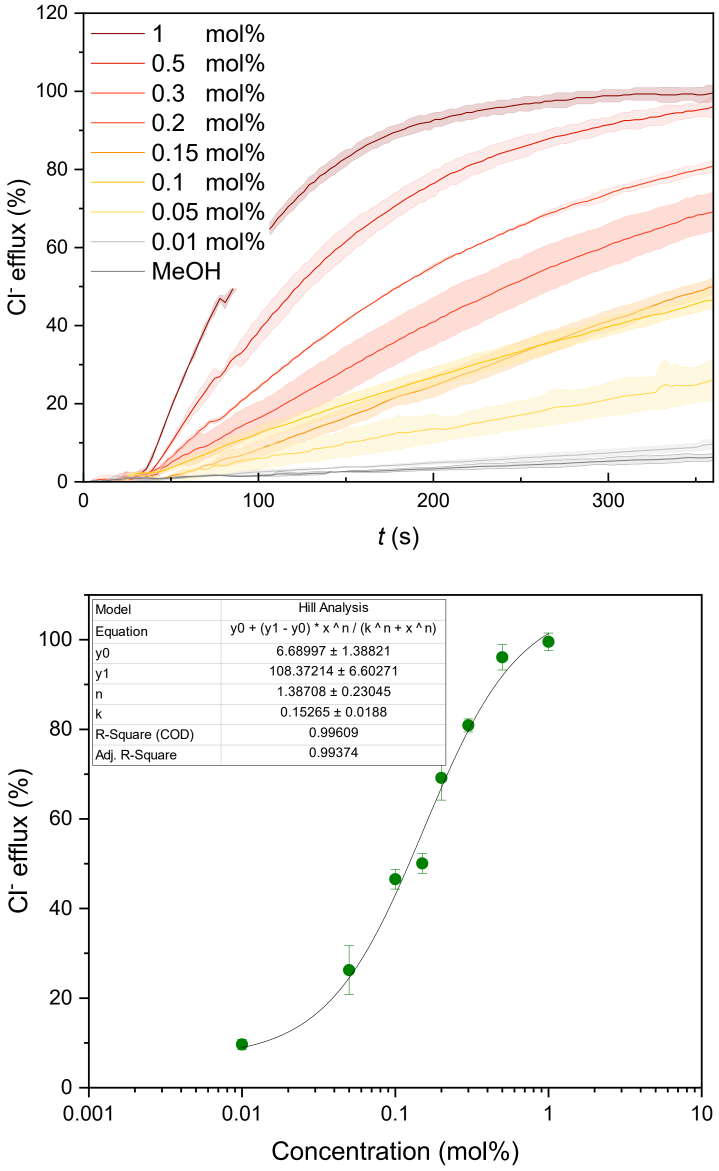

Figure S31. (Top) Chloride efflux plotted versus time as measured by a chloride selective electrode in the monensin-coupled assay. The transporter was added from MeOH (1.0 mM stock solution) to the aqueous liposome solution (1.0 mM POPC, 300 mM internal KCl, 300 mM external KGlu buffered with MES to pH 5.5) at t = 15 s, followed by monensin (0.1 mol%) from DMSO at t = 30 s, and an aqueous Triton-X solution at t = 360 s. Blank experiments were also performed in which MeOH (without transporter) was added. (Bottom) Chloride efflux at t = 360 s in the monensin-coupled ISE assay plotted versus concentration and fit to the Hill equation. The same data is shown in Figure 3B of the main text without details of the fit; EC_{50} = 1.5×10^{-1} mol% (with respect to lipids).

Comparison of (*Z*)-1 and (*E*PSS)-1 at pH 7.2

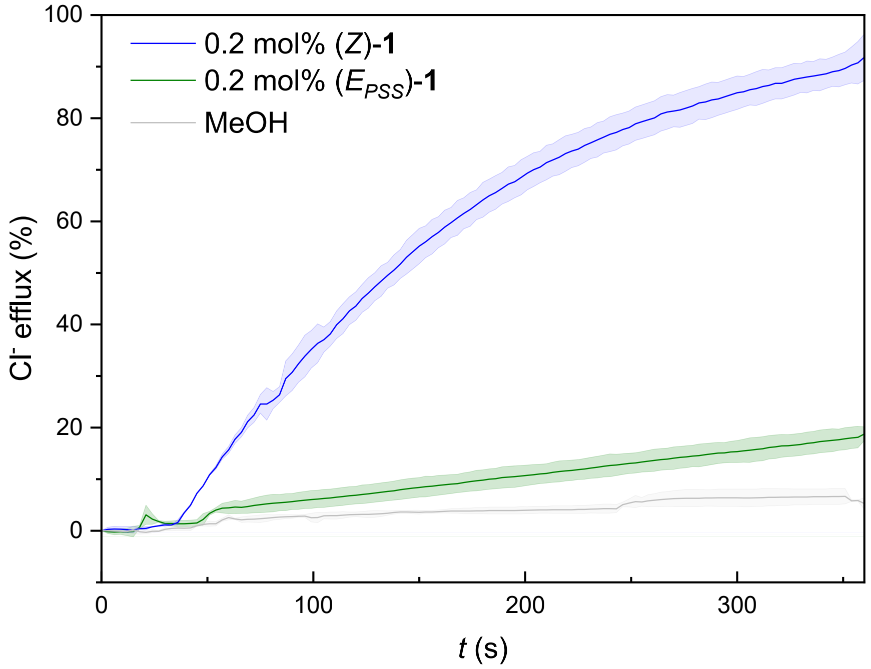

Figure S32. Chloride efflux plotted versus time as measured by a chloride selective electrode in the monensin-coupled assay. The transporters were added from MeOH (1.0 mM stock solution) to the aqueous liposome solution (1.0 mM POPC, 300 mM internal KCl, 300 mM external KGlu buffered with HEPES to pH 7.2) at t = 15 s, followed by monensin (0.1 mol%) from DMSO at t = 30 s, and an aqueous Triton-X solution at t = 360 s. Blank experiments were also performed in which MeOH (without transporter) was added.

Comparison of (*Z*)-1 and (E_{PSS})-1 at pH 5.5

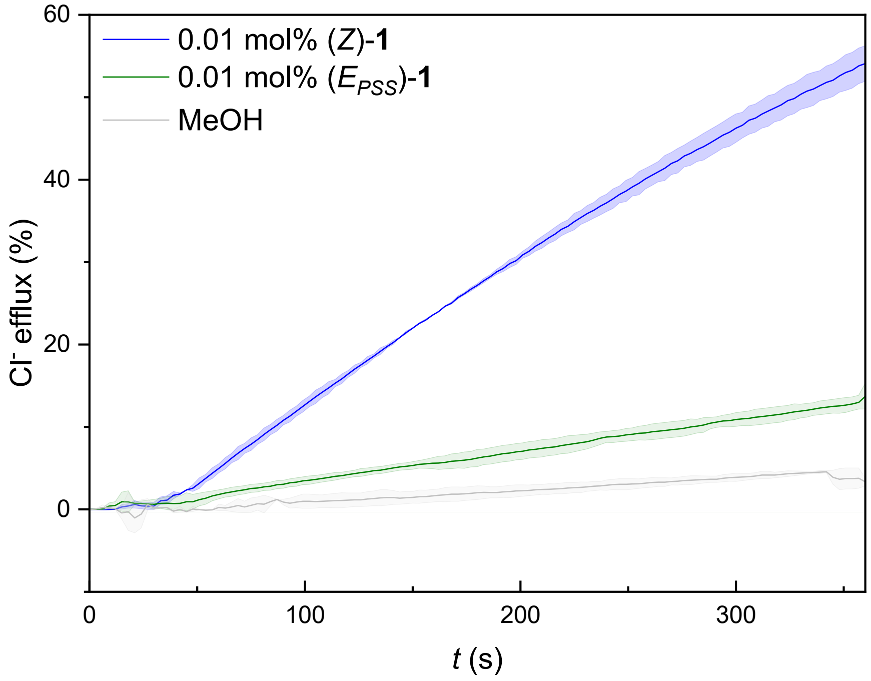

Figure S33. Chloride efflux plotted versus time as measured by a chloride selective electrode in the monensin-coupled assay. The transporters were added from MeOH (1.0 mM stock solution) to the aqueous liposome solution (1.0 mM POPC, 300 mM internal KCl, 300 mM external KGlu buffered with MES to pH 5.5) at *t* = 15 s, followed by monensin (0.1 mol%) from DMSO at *t* = 30 s, and an aqueous Triton-X solution at *t* = 360 s. Blank experiments were also performed in which MeOH (without transporter) was added.

In-situ photodeactivation of (*Z*)-1 at pH 7.2

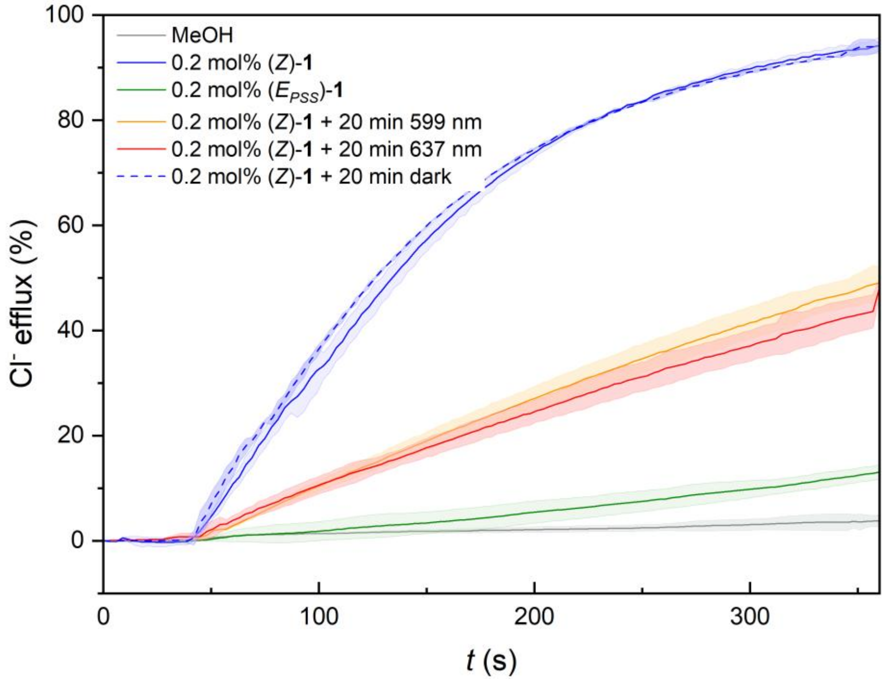

Figure S34. Chloride efflux plotted versus time as measured by a chloride selective electrode in the monensin-coupled assay. The transporter was added from MeOH (1.0 mM stock solution) to the aqueous liposome solution (1.0 mM POPC, 300 mM internal KCl, 300 mM external KGlu buffered with HEPES to pH 7.2) at t = 15 s, followed by monensin (0.1 mol%) from DMSO at t = 30 s, and an aqueous Triton-X solution at t = 360 s. For the *in situ* irradiated samples the transporter was added to the liposome prior to assay, and irradiated for 20 minutes. An identical sample was prepared and kept in the dark for the same amount of time. Blank experiments were also performed in which MeOH (without transporter) was added. Irradiation times were based on the kinetic measurements by UV-Vis spectroscopy shown in Figure S42.

In-situ photodeactivation of (*Z*)-1 at pH = 5.5

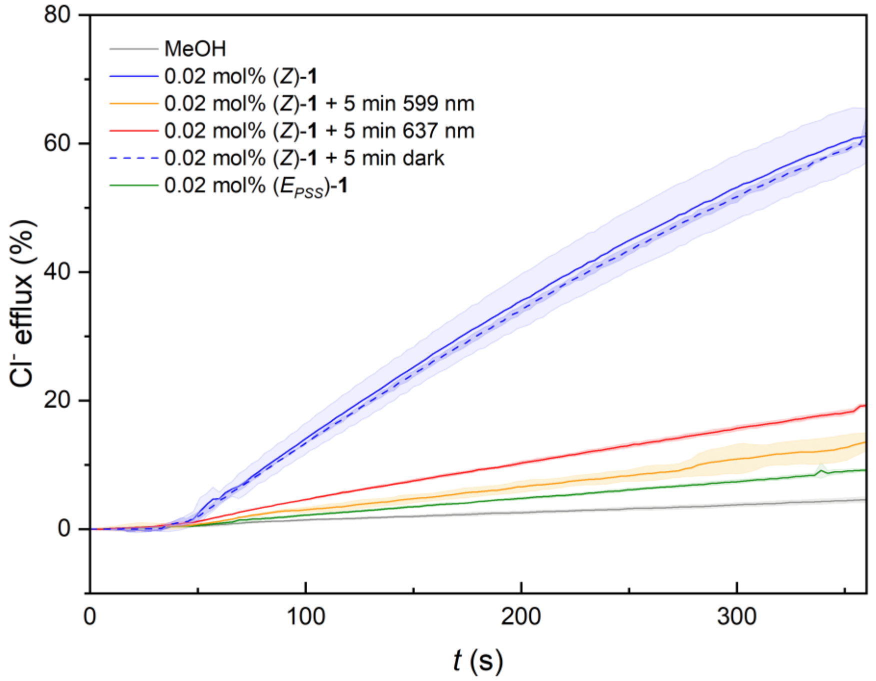

Figure S35. Chloride efflux plotted versus time as measured by a chloride selective electrode in the monensin-coupled assay. The transporter was added from MeOH (1.0 mM stock solution) to the aqueous liposome solution (1.0 mM POPC, 300 mM internal KCl, 300 mM external KGlu buffered with MES to pH 5.5) at t = 15 s, followed by monensin (0.1 mol%) from DMSO at t = 30 s, and an aqueous Triton-X solution at t = 360 s. For the *in situ* irradiated samples the transporter was added to the liposome prior to assay, and irradiated for 5 minutes. An identical sample was prepared and kept in the dark for the same amount of time. Blank experiments were also performed in which MeOH (without transporter) was added. Irradiation times were based on the kinetic measurements by UV-Vis spectroscopy shown in Figure S41.

7. UV-Vis studies in POPC liposomes

Comparison of (*Z*/*E*)-1 in MeOH and POPC liposomes at different pH

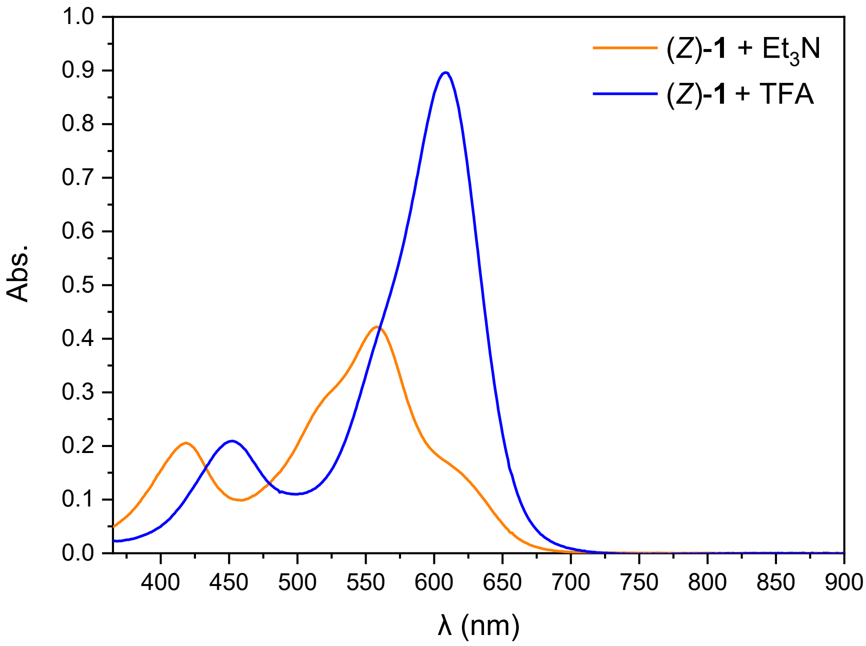

Figure S36. UV-Vis spectra of as-isolated (*Z*)-**1**·TFA dissolved in MeOH (15 µM, 273 K) in presence of either 3 equiv. Et_3N or 3 equiv. TFA.

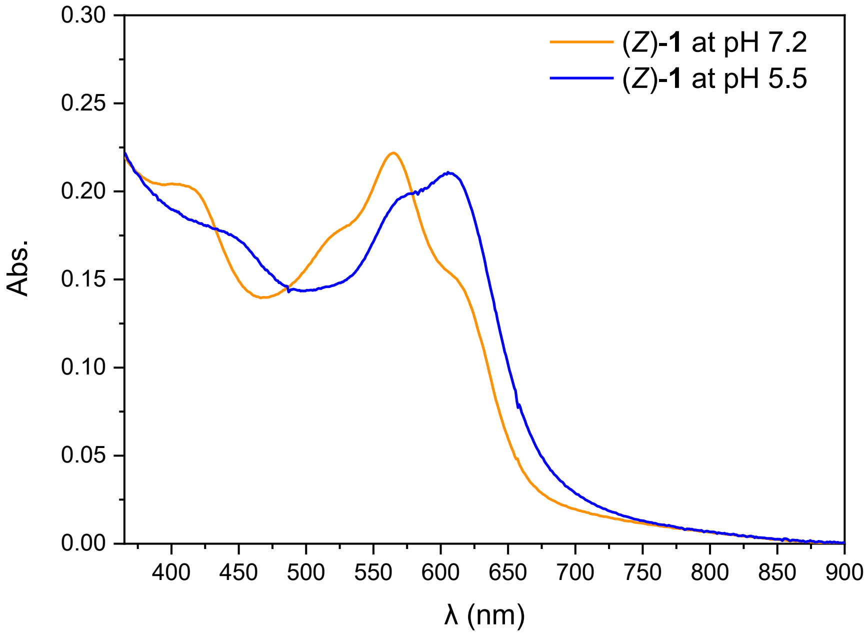

Figure S37. UV-Vis spectra of 1 mol% (*Z*)-**1** in POPC liposomes (1 mM POPC, 300 mM KGlu external solution and 300 mM KCl internal solution) prepared using either 5 mM MES buffer at pH 5.5 or 5 mM HEPES buffer at pH 7.2.

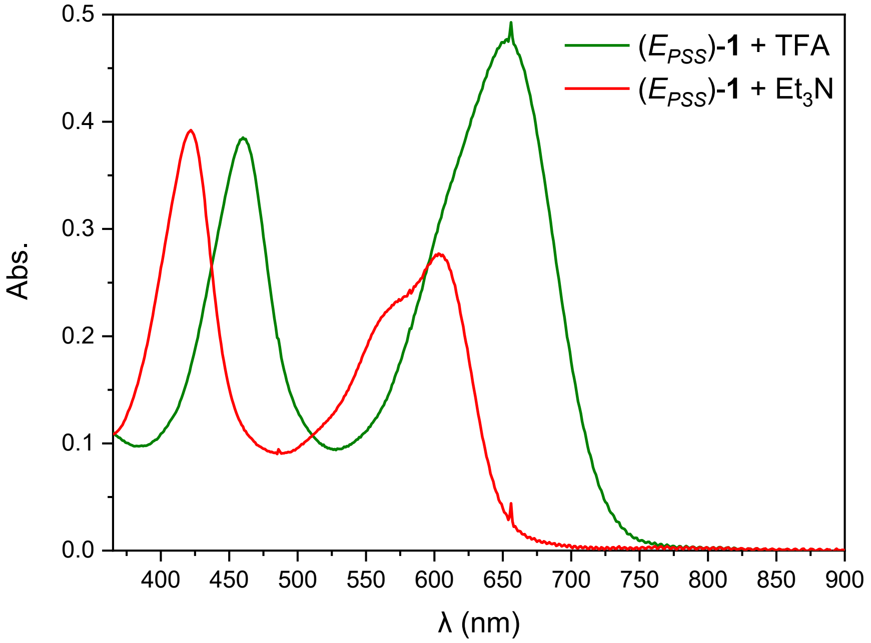

Figure S38. Photogenerated (E_{PSS})-**1**·H$^+$ (PSS 599 nm) in MeOH (15 µM, 3 equiv. TFA, 273 K) neutralized with Et_3N (10 equiv.).

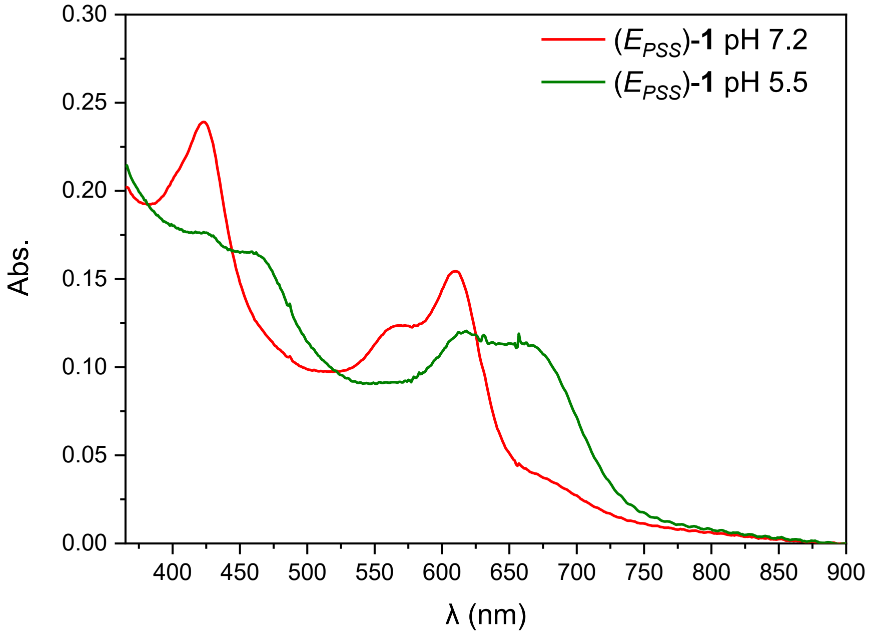

Figure S39. UV-Vis spectra of 1 mol% (E_{PSS})-**1** (pre-generated photochemically from a 1 mM solution in MeOH by irradiation with 599 nm light) in POPC liposomes (1 mM POPC, 300 mM KGlu external solution and 300 mM KCl internal solution) prepared using either 5 mM MES buffer at pH = 5.5 or 5 mM HEPES buffer at pH = 7.2.

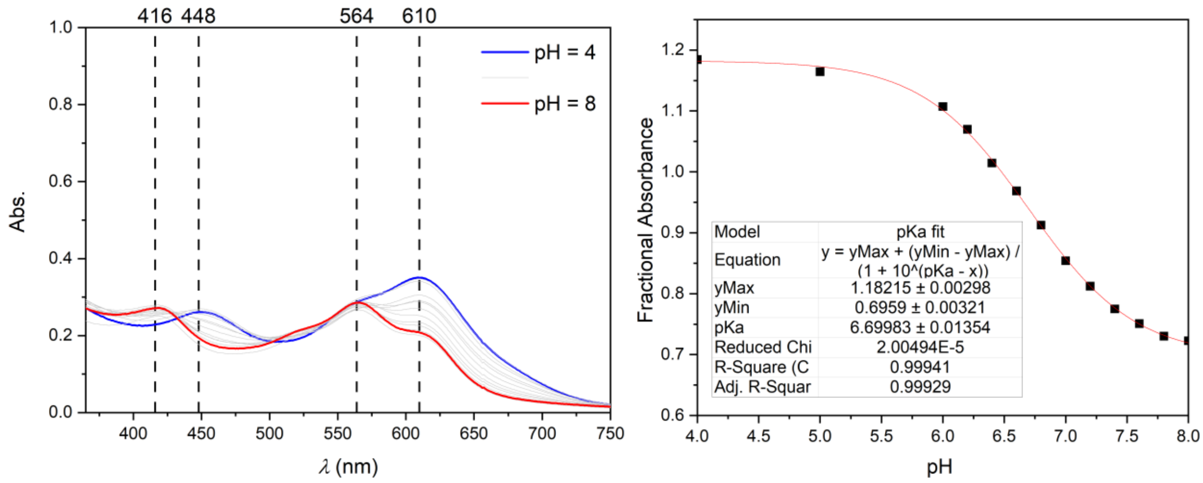

Figure S40. (Left) UV-Vis spectra of POPC liposomes (1 mM, 300 mM KCl, 5 mM MES buffer pH 5.5) dissolved in an external solution with varying pH (10 mM citrate buffer from pH 4 to pH 5, 10 mM phosphate buffer for pH 6 to pH 8, with 300 mM KCl) to which 1 mol % (*Z*)-**1** was added from a MeOH solution (1 mM). Each spectrum is an individually prepared sample. (Right) Fractional absorbance [(Abs$_{610}$+Abs$_{448}$)/(Abs$_{416}$+Abs$_{564}$)] followed over the indicated pH range and fitted to a sigmoidal function which affords an apparent pK_a value of 6.7.

Photoisomerization in POPC liposomes

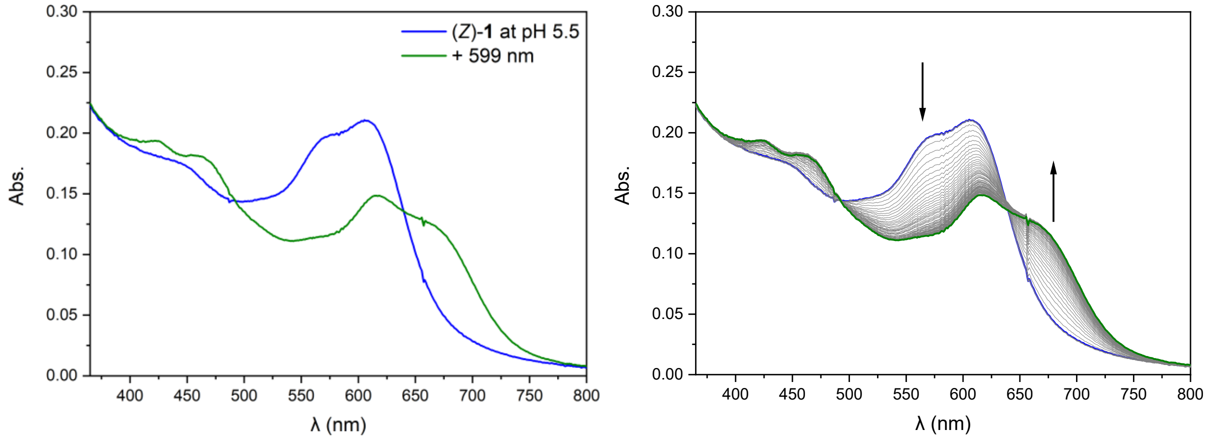

Figure S41. (Left) UV-Vis spectra of 1 mol% (*Z*)- **1** in POPC liposomes (1 mM POPC, 10 µM (*Z*)-**1** in 5 mM MES buffer pH = 5.5, 300 mM KGlu external solution and 300 mM KCl internal solution) before and after irradiation with 599 nm light. (Right) Spectral changes followed over time (5 s interval) upon irradiation with 599 nm light.

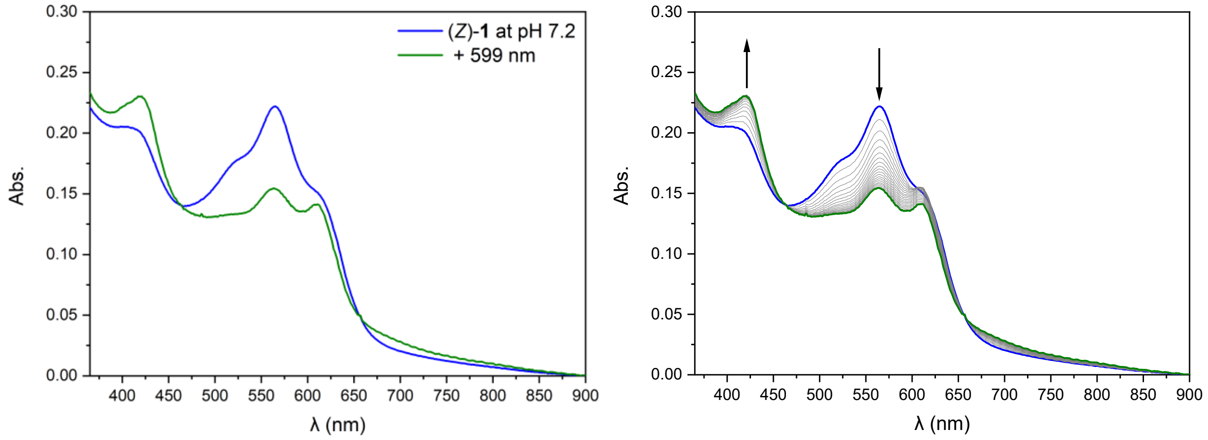

Figure S42. (Left) UV-Vis spectra of 1 mol% (*Z*)- **1** in POPC liposomes (1 mM POPC, 10 µM (*Z*)-**1** in 5 mM HEPES buffer pH = 7.2, 300 mM KGlu external solution and 300 mM KCl internal solution) before and after irradiation with 599 nm light. (Right) Spectral changes followed over time (60 s interval) upon irradiation with 599 nm light.

8. DFT Calculations

All calculations were performed using Gaussian 16[4], and data was visualized using Avogadro 1.2.0[5] and Gaussview 6[6]. Initial conformational searches were performed using CREST[7]. The lowest energy conformers were subsequently optimized at the B3LYP-D3/cc-pVDZ level of theory (IEFPCM MeOH), followed by the B3LYP-D3/aug-cc-pVTZ level of theory (IEFPCM MeOH). All computed stationary points were subjected to frequency analysis and confirmed as local minima (no imaginary frequencies found for all minima).

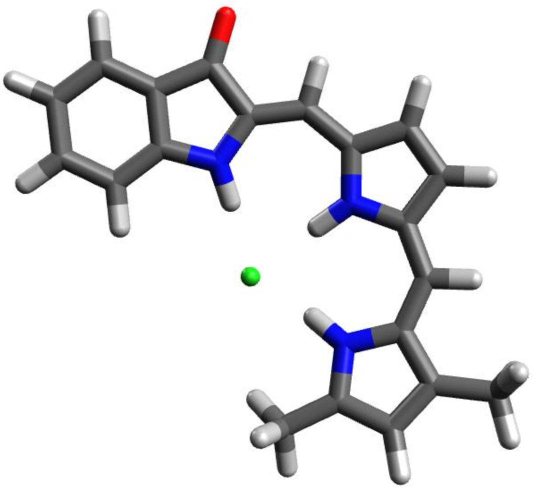

Figure S43. Optimized geometry of (*Z*)-**1**·HCl calculated at the B3LYP-D3/aug-cc-pVTZ level of theory with an IEFPCM MeOH solvent model.

Zero-point correction =	0.338494 (Hartree/Particle)
Thermal correction to Energy =	0.361100
Thermal correction to Enthalpy =	0.362044
Thermal correction to Gibbs Free Energy =	0.283647
Sum of electronic and zero-point Energies =	–1472.817642
Sum of electronic and thermal Energies =	–1472.795035
Sum of electronic and thermal Enthalpies =	–1472.794091
Sum of electronic and thermal Free Energies =	–1472.872488

Atom	X	Y	Z
Cl	-0.2773710000	-1.6895410000	1.2053640000
O	4.4773250000	2.4559570000	-0.0574050000
N	2.1575690000	-0.1632970000	-0.0638550000
N	-3.0834160000	-1.1030120000	-0.0242190000
N	-0.8114490000	1.3921180000	0.1489420000
C	5.9537030000	-1.8048810000	-0.3973040000
C	4.8639140000	-2.6852750000	-0.3526720000
C	3.5529690000	-2.2326000000	-0.2377950000
C	3.3573380000	-0.8594940000	-0.1665480000
C	4.4405720000	0.0310520000	-0.2001760000
C	5.7459800000	-0.4347970000	-0.3191900000
C	2.3960670000	1.1818070000	0.0328930000
C	3.8965500000	1.3810220000	-0.0726120000
C	1.5595350000	2.2423950000	0.1522560000
C	-3.9033690000	-2.1518500000	-0.1748940000
C	-5.2097080000	-1.6678280000	-0.3843670000
C	-5.1695060000	-0.2874400000	-0.3382930000
C	-3.8044680000	0.0843190000	-0.1066660000
C	-3.3327460000	1.3781780000	-0.0319280000
C	-2.0712910000	1.9694480000	0.0732750000
C	-1.8739280000	3.3708010000	0.0346990000
C	-0.5227350000	3.6164320000	0.0876830000
C	0.1427630000	2.3694130000	0.1549720000
C	-3.4170780000	-3.5528460000	-0.1304820000
C	-6.3152390000	0.6511050000	-0.5123990000
H	6.9554560000	-2.1991590000	-0.4900020000
H	5.0455440000	-3.7500740000	-0.4101050000
H	2.7204030000	-2.9202340000	-0.2044120000
H	6.5731770000	0.2614010000	-0.3443110000
H	1.3011800000	-0.6334450000	0.2341960000
H	2.0785160000	3.1917640000	0.1912690000

H	-2.1074440000	-1.2099980000	0.2709450000
H	-6.0775800000	-2.2840980000	-0.5478630000
H	-4.1178810000	2.1198550000	-0.1051480000
H	-0.6326720000	0.4091170000	0.3454980000
H	-2.6700770000	4.0918150000	-0.0455620000
H	-0.0236550000	4.5697650000	0.0584200000
H	-4.1957080000	-4.2141880000	0.2439530000
H	-2.5292710000	-3.6391720000	0.4938900000
H	-3.1514570000	-3.8885970000	-1.1361720000
H	-7.2413970000	0.0992800000	-0.6574620000
H	-6.1683310000	1.3001040000	-1.3774240000
H	-6.4369570000	1.2959690000	0.3595530000

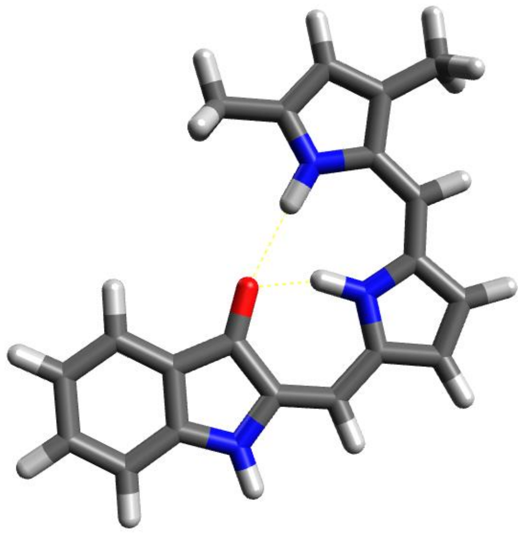

Figure S44. Optimized geometry of (*E*)-**1**·H^+ calculated at B3LYP-D3/aug-cc-pVTZ level of theory with an IEFPCM MeOH solvent model.

Zero-point correction =	0.338341 (Hartree/Particle)
Thermal correction to Energy =	0.358937
Thermal correction to Enthalpy =	0.359881
Thermal correction to Gibbs Free Energy =	0.287341
Sum of electronic and zero-point Energies =	–1012.382928
Sum of electronic and thermal Energies =	–1012.362331
Sum of electronic and thermal Enthalpies =	–1012.361387
Sum of electronic and thermal Free Energies =	–1012.433927

Atom	X	Y	Z
O	-0.7211130000	-0.5681620000	0.0048440000
N	2.3449540000	-1.2083300000	0.0011740000
N	0.8577940000	1.5437130000	0.0023990000
N	-3.5635620000	1.4992240000	-0.0018380000
C	5.9750820000	-0.4528330000	-0.0066880000
C	1.9796260000	-3.6553310000	0.0074750000
C	2.8474670000	-2.4535540000	-0.0018460000
C	4.2499450000	-2.3616220000	-0.0057380000
C	4.5983650000	-1.0234200000	-0.0036340000

C	3.3818260000	-0.2690510000	0.0005770000
C	3.2842280000	1.1071630000	0.0012940000
C	2.1683170000	1.9456710000	0.0021740000
C	2.1552330000	3.3657600000	0.0014980000
C	0.8398750000	3.7797660000	0.0010660000
C	0.0300200000	2.6150470000	0.0015350000
C	-1.3865680000	2.5322040000	0.0004870000
C	-1.8483570000	-0.0489210000	0.0021910000
C	-2.1828950000	1.4235260000	0.0002270000
C	-3.4346460000	-2.0898980000	0.0010770000
C	-3.1210560000	-0.7294430000	0.0006340000
C	-4.1343340000	0.2459060000	-0.0019850000
C	-5.4777510000	-0.1135340000	-0.0042780000
C	-5.7714570000	-1.4708090000	-0.0038380000
C	-4.7685200000	-2.4556890000	-0.0011860000
H	6.1415180000	0.1721090000	-0.8855420000
H	6.7174260000	-1.2475330000	-0.0090310000
H	6.1458570000	0.1709750000	0.8721430000
H	1.2615590000	-3.6210210000	-0.8135050000
H	1.4089710000	-3.7067460000	0.9372080000
H	2.5782330000	-4.5572760000	-0.0832980000
H	1.3528990000	-1.0291400000	0.0042680000
H	4.9205810000	-3.2038330000	-0.0101530000
H	4.2323200000	1.6281790000	0.0005800000
H	0.4575470000	0.6020990000	0.0032100000
H	3.0367320000	3.9843440000	0.0011520000
H	0.4684410000	4.7899640000	0.0002920000
H	-1.8903960000	3.4908490000	-0.0007150000
H	-4.0812060000	2.3615250000	-0.0039650000
H	-2.6458660000	-2.8291570000	0.0031090000
H	-6.2608790000	0.6306380000	-0.0063480000
H	-6.8080910000	-1.7792330000	-0.0056080000
H	-5.0474370000	-3.4993300000	-0.0009550000

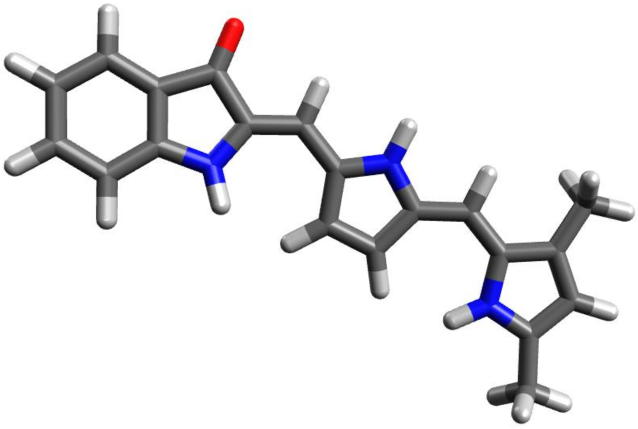

Figure S45. The optimized geometry of (*Z*)-**1**·H$^+$ calculated at B3LYP GD3 aug-cc-pVTZ level of theory with an IEFPCM MeOH solvent model. Color codes: O = red; N = blue.

Zero-point correction= 0.338115 (Hartree/Particle)

Thermal correction to Energy= 0.359220

Thermal correction to Enthalpy= 0.360164

Thermal correction to Gibbs Free Energy= 0.285053

Sum of electronic and zero-point Energies= -1012.373029

Sum of electronic and thermal Energies= -1012.351924

Sum of electronic and thermal Enthalpies= -1012.350980

Sum of electronic and thermal Free Energies= -1012.426091

Atom	X	Y	Z
O	-4.0840080000	-2.6032640000	0.1321850000
N	4.7704930000	1.0794640000	0.0256170000
N	0.8731770000	-1.1825940000	0.0065080000
N	-3.1643740000	0.7630450000	0.0056570000
C	5.9997650000	-2.4082360000	-0.1836120000
C	6.6777930000	2.6636080000	0.0308400000
C	6.0961910000	1.3004930000	-0.0032810000
C	6.7412820000	0.0548390000	-0.0815190000
C	5.7760410000	-0.9371050000	-0.1058890000

C	4.5041680000	-0.2862920000	-0.0363240000
C	3.2699440000	-0.8985090000	-0.0302040000
C	1.9851270000	-0.3582240000	0.0558440000
C	1.4811920000	0.9558080000	0.2026170000
C	0.1045420000	0.8919390000	0.2304630000
C	-0.2755750000	-0.4680510000	0.1016410000
C	-1.5433390000	-1.1129750000	0.0818410000
C	-4.0396460000	-1.3852580000	0.0702600000
C	-2.7744100000	-0.5513270000	0.0540940000
C	-6.5099540000	-0.5729380000	-0.0265490000
C	-5.1285800000	-0.4126790000	0.0006270000
C	-4.5522480000	0.8648770000	-0.0507450000
C	-5.3352320000	2.0061210000	-0.1369310000
C	-6.7162360000	1.8308250000	-0.1659800000
C	-7.3054810000	0.5613790000	-0.1100460000
H	5.5845550000	-2.9173700000	0.6875940000
H	7.0631520000	-2.6304400000	-0.2323330000
H	5.5216190000	-2.8342180000	-1.0671540000
H	6.2859370000	3.2321100000	0.8754580000
H	6.4222030000	3.2100340000	-0.8796120000
H	7.7599490000	2.6116560000	0.1109440000
H	4.0825180000	1.8100270000	0.0662310000
H	7.8084810000	-0.0822620000	-0.1165300000
H	3.3139410000	-1.9780660000	-0.1065580000
H	0.9101430000	-2.1834010000	-0.1063030000
H	2.0575150000	1.8580660000	0.2973620000
H	-0.5551480000	1.7296480000	0.3628780000
H	-1.5449540000	-2.1958980000	0.0862940000
H	-2.5538530000	1.5435870000	-0.1522500000
H	-6.9438500000	-1.5624790000	0.0141610000
H	-4.8942070000	2.9916030000	-0.1789250000
H	-7.3512670000	2.7037410000	-0.2326820000
H	-8.3818030000	0.4705850000	-0.1334040000

----

9. References

(1) T. Arai, M. Ikegami, *Chem. Lett.* **1999**, *28*, 965–966.

(2) C. Frassineti, S. Ghelli, P. Gans, A. Sabatini, M. S. Moruzzi, A. Vacca, *Anal. Biochem.* **1995**, *231*, 374–382.

(3) L. A. Jowett, E. N. W. Howe, V. Soto-Cerrato, W. Van Rossom, R. Pérez-Tomás, P. A. Gale, *Sci. Rep.* **2017**, *7*, 9397.

(4) Gaussian 16, Revision C.01, M. J. Frisch, G. W. Trucks, H. B. Schlegel, G. E. Scuseria, M. A. Robb, J. R. Cheeseman, G. Scalmani, V. Barone, G. A. Petersson, H. Nakatsuji, X. Li, M. Caricato, A. V. Marenich, J. Bloino, B. G. Janesko, R. Gomperts, B. Mennucci, H. P. Hratchian, J. V. Ortiz, A. F. Izmaylov, J. L. Sonnenberg, D. Williams-Young, F. Ding, F. Lipparini, F. Egidi, J. Goings, B. Peng, A. Petrone, T. Henderson, D. Ranasinghe, V. G. Zakrzewski, J. Gao, N. Rega, G. Zheng, W. Liang, M. Hada, M. Ehara, K. Toyota, R. Fukuda, J. Hasegawa, M. Ishida, T. Nakajima, Y. Honda, O. Kitao, H. Nakai, T. Vreven, K. Throssell, J. A. Montgomery, Jr., J. E. Peralta, F. Ogliaro, M. J. Bearpark, J. J. Heyd, E. N. Brothers, K. N. Kudin, V. N. Staroverov, T. A. Keith, R. Kobayashi, J. Normand, K. Raghavachari, A. P. Rendell, J. C. Burant, S. S. Iyengar, J. Tomasi, M. Cossi, J. M. Millam, M. Klene, C. Adamo, R. Cammi, J. W. Ochterski, R. L. Martin, K. Morokuma, O. Farkas, J. B. Foresman, and D. J. Fox, Gaussian, Inc., Wallingford CT, 2016.

(5) Avogadro: an open-source molecular builder and visualization tool. Version 1.2.0 http://avogadro.cc/

(6) GaussView, Version 6, R. Dennington, T. A. Keith, J. M. Millam, Semichem Inc., Shawnee Mission, KS, 2016.

(7) P. Pracht, F. Bohle, S. Grimme, *Phys. Chem. Chem. Phys.* **2020**, *22*, 7169–7192.
